# Local sequence alignments statistics: deviations from Gumbel statistics in the rare-event tail

**DOI:** 10.1186/1748-7188-2-9

**Published:** 2007-07-11

**Authors:** Stefan Wolfsheimer, Bernd Burghardt, Alexander K Hartmann

**Affiliations:** 1 Institut für Theoretische Physik, Universität Göttingen, 37077, Göttingen, Friedrich-Hund-Platz 1, Germany; 2 Institut für Physik, Universität Oldenburg, 26111, Oldenburg, Germany

## Abstract

**Background:**

The optimal score for ungapped local alignments of infinitely long random sequences is known to follow a Gumbel extreme value distribution. Less is known about the important case, where gaps are allowed. For this case, the distribution is only known empirically in the high-probability region, which is biologically less relevant.

**Results:**

We provide a method to obtain numerically the biologically relevant rare-event tail of the distribution. The method, which has been outlined in an earlier work, is based on generating the sequences with a parametrized probability distribution, which is biased with respect to the original biological one, in the framework of Metropolis Coupled Markov Chain Monte Carlo. Here, we first present the approach in detail and evaluate the convergence of the algorithm by considering a simple test case. In the earlier work, the method was just applied to one single example case. Therefore, we consider here a large set of parameters:

We study the distributions for protein alignment with different substitution matrices (BLOSUM62 and PAM250) and affine gap costs with different parameter values. In the logarithmic phase (large gap costs) it was previously assumed that the Gumbel form still holds, hence the Gumbel distribution is usually used when evaluating p-values in databases. Here we show that for all cases, provided that the sequences are not too long (*L *> 400), a "modified" Gumbel distribution, i.e. a Gumbel distribution with an additional Gaussian factor is suitable to describe the data. We also provide a "scaling analysis" of the parameters used in the modified Gumbel distribution. Furthermore, via a comparison with BLAST parameters, we show that significance estimations change considerably when using the true distributions as presented here. Finally, we study also the distribution of the sum statistics of the *k *best alignments.

**Conclusion:**

Our results show that the statistics of gapped and ungapped local alignments deviates significantly from Gumbel in the rare-event tail. We provide a Gaussian correction to the distribution and an analysis of its scaling behavior for several different scoring parameter sets, which are commonly used to search protein data bases. The case of sum statistics of *k *best alignments is included.

## Background

Sequence alignment is a powerful tool in bioinformatics [1,2] to detect evolutionarily related proteins by comparing their sequences of amino acids. Basically one wants to determine the "similarity" of the sequences. For example, given a protein in a database like PDB [3], such similarity analysis can be used to detect other proteins, which are evolutionary close to it. Related approaches are also used for the comparison of DNA sequences, i.e. shotgun DNA sequencing [4], but the application to DNA is not considered in this article.

Alignment algorithms find optimum alignments and maximum alignment scores *S *of two or more sequences for a given scoring system. Needleman and Wunsch suggested a method to compute global alignments [5], whereas the Smith-Waterman algorithm [6] aims at finding local similarities. Insertions and deletions of residues are taken into account by allowing for gaps in the alignment. Gaps yield a negative contribution to the alignment score and are usually modeled by a gap-length *l *depending score function *g *(*l*). Widely used are affine gap costs because for two given sequences of length *L *and *M*, because fast algorithms with running time O
 MathType@MTEF@5@5@+=feaafiart1ev1aaatCvAUfKttLearuWrP9MDH5MBPbIqV92AaeXatLxBI9gBamrtHrhAL1wy0L2yHvtyaeHbnfgDOvwBHrxAJfwnaebbnrfifHhDYfgasaacH8akY=wiFfYdH8Gipec8Eeeu0xXdbba9frFj0=OqFfea0dXdd9vqai=hGuQ8kuc9pgc9s8qqaq=dirpe0xb9q8qiLsFr0=vr0=vr0dc8meaabaqaciaacaGaaeqabaWaaeGaeaaakeaaimaacqWFoe=taaa@383D@ (*LM*) are available for this case [7]. Note that for database queries even this is too complex, hence fast heuristics like BLAST [8] are used there.

By itself, the alignment *score*, which measures the similarity of two given sequences, does not contain any information about the statistical significance of an alignment. One approach to quantify the statistical significance is to compute the *p-value *for a given score *S*. This means under a random sequence model one wants to know the probability for the occurrence of at least one hit with a score *S *greater than or equal to some given threshold value *b*, i.e. ℙ(*S *≥ *b*). Often E-values are used instead. They describe the number of expected hits with a score greater than or equal to some threshold value. One possible access to the statistical significance can be achieved under the null model of random sequences. Then the optimal alignment score *S *becomes a random variable and the probability of occurrence of *S *under this model *P *(*s*) = ℙ (*S *= *s*) provides estimates for p-values. Analytic expressions for *P *(*s*) are only known asymptotically in the case of gapless alignments of long sequences, where an *extreme value distribution *(also called *Gumbel distribution*) [9,10] was found. For alignments with gaps, such analytical expressions are not available. Approximation for scenarios with gaps based on probabilistic alignment [11-13], large deviations [14] and a Poisson model [15] had been developed. Altschul and Gish [16] investigated the score statistics of random sequences for a number of scoring systems and gap parameters by computer simulations: They obtained histograms of optimum scores for randomly sampled pairs of sequences by simple sampling. By curve fitting, they showed that in the region of high probability the extreme value distribution describes the data well, also for gapped alignments of finite sequences. Additionally, they found that the theoretical predictions for the relation between the scoring system on one side and the Gumbel parameters on the other side hold approximately for gapped alignments. In this context they obtained two improvements: Using a correction to account for finite sequence lengths and sum statistics of the *k*-best alignments, theoretical predictions for ungapped alignments could be applied more accurately to gapped alignments. Recently Olsen et al. introduced the "island method" [17,18], which accelerates sampling time. BLAST [8] uses precomputed data, generated with the island method, to estimate E-values. In any case, as already pointed out, the studies in Ref. [16] and [18] give reliable data in the region where *P *(*s*) is large only. This is outside the region of biological interest because pairs of biologically related sequences have a higher similarity than pairs of purely randomly drawn sequences.

To overcome this drawback a rare-event sampling technique was proposed recently [19], which is based on methods from statistical physics. This general approach allows to obtain the distribution over a wide range, in the present case down to *P *(*s*) = 10^-40^. So far this method has been applied to one relevant case only, namely protein alignment with the BLOSUM 62 score matrix [7] and affine gap costs with *α *= 12 opening and *β *= 1 extension costs. It turned out that at least for one scoring matrix and one set of gap-cost parameters, the distribution deviates from the Gumbel form in the biologically relevant rare-event tail, where simple sampling methods fail. Empirically, a Gaussian correction to the original distribution was proposed for this case.

Results as in Ref. [19] are only useful if one obtains the distribution for a large range of parameter values which are commonly used in bioinformatics. It is the purpose of this work to study the distribution of *S *for other relevant cases. Here we consider the BLOSUM62 and the PAM250 score matrices in connection with various parameters *α *, *β *of affine gap costs.

The paper is organized as follows. In the second section we define alignments formally and state a few main results on the statistics of local sequence alignment. Next, we state the rare-event approach used here and in the fourth section we explain our approach in detail. We introduce some toy examples which are also used to evaluate the convergence properties of the algorithm. In the fifth section, we present our results for BLOSUM62 and PAM 250 matrices in conjunction with different affine gap costs. We show also our results for the sum statistics of the *k *largest alignments. In the last section, we summarize and discuss our results.

## Statistics of local sequence alignment

In this section, we define sequence alignment, and state some analytical results for the distribution of the optimum scores *S *over pairs of random sequences.

Let **x **= *x*_1_*x*_2 _... *x*_*L *_and **y **= *y*_1_*y*_2 _... *y*_*M *_be two sequences over a finite alphabet Σ with *r *= |Σ| letters(e.g. nucleic acids or amino acids). An *alignment *A
 MathType@MTEF@5@5@+=feaafiart1ev1aaatCvAUfKttLearuWrP9MDH5MBPbIqV92AaeXatLxBI9gBamrtHrhAL1wy0L2yHvtyaeHbnfgDOvwBHrxAJfwnaebbnrfifHhDYfgasaacH8akY=wiFfYdH8Gipec8Eeeu0xXdbba9frFj0=OqFfea0dXdd9vqai=hGuQ8kuc9pgc9s8qqaq=dirpe0xb9q8qiLsFr0=vr0=vr0dc8meaabaqaciaacaGaaeqabaWaaeGaeaaakeaaimaacqWFaeFqaaa@3821@ is a set A
 MathType@MTEF@5@5@+=feaafiart1ev1aaatCvAUfKttLearuWrP9MDH5MBPbIqV92AaeXatLxBI9gBamrtHrhAL1wy0L2yHvtyaeHbnfgDOvwBHrxAJfwnaebbnrfifHhDYfgasaacH8akY=wiFfYdH8Gipec8Eeeu0xXdbba9frFj0=OqFfea0dXdd9vqai=hGuQ8kuc9pgc9s8qqaq=dirpe0xb9q8qiLsFr0=vr0=vr0dc8meaabaqaciaacaGaaeqabaWaaeGaeaaakeaaimaacqWFaeFqaaa@3821@ = {(*i*_*k*_, *j*_*k*_} of *K *pairs of "non-crossing" indices (*k *= 1, 2, ..., *K *- 1, 1 ≤ *i*_*k *_<*i*_*k*+1 _≤ *L *and 1 ≤ *j*_*k *_<*j*_*k*+1 _≤ *M*) identifying pairs of letters from the two sequences. Letters, which are not paired are called *unpaired *or *gapped*. A *gap g of length l*_*g *_is a substring of *l*_*g *_gapped letters from one sequence. Note, that this representation [14] of an alignment is equivalent to an introduction of a gap symbol, as commonly used. Formally the gap cost function can be defined by considering the length of a gap beginning at the *k*th pairing in sequence **x **or sequence **y **respectively, in detail

lgx(k)=ik+1−ik−1lgy(k)=jk+1−jk−1.
 MathType@MTEF@5@5@+=feaafiart1ev1aaatCvAUfKttLearuWrP9MDH5MBPbIqV92AaeXatLxBI9gBaebbnrfifHhDYfgasaacH8akY=wiFfYdH8Gipec8Eeeu0xXdbba9frFj0=OqFfea0dXdd9vqai=hGuQ8kuc9pgc9s8qqaq=dirpe0xb9q8qiLsFr0=vr0=vr0dc8meaabaqaciaacaGaaeqabaqabeGadaaakeaafaqabeGabaaabaGaemiBaW2aa0baaSqaaiabdEgaNbqaaiabdIha4baakiabcIcaOiabdUgaRjabcMcaPiabg2da9iabdMgaPnaaBaaaleaacqWGRbWAcqGHRaWkcqaIXaqmaeqaaOGaeyOeI0IaemyAaK2aaSbaaSqaaiabdUgaRbqabaGccqGHsislcqaIXaqmaeaacqWGSbaBdaqhaaWcbaGaem4zaCgabaGaemyEaKhaaOGaeiikaGIaem4AaSMaeiykaKIaeyypa0JaemOAaO2aaSbaaSqaaiabdUgaRjabgUcaRiabigdaXaqabaGccqGHsislcqWGQbGAdaWgaaWcbaGaem4AaSgabeaakiabgkHiTiabigdaXiabc6caUaaaaaa@5399@

The *score *S˜
 MathType@MTEF@5@5@+=feaafiart1ev1aaatCvAUfKttLearuWrP9MDH5MBPbIqV92AaeXatLxBI9gBaebbnrfifHhDYfgasaacH8akY=wiFfYdH8Gipec8Eeeu0xXdbba9frFj0=OqFfea0dXdd9vqai=hGuQ8kuc9pgc9s8qqaq=dirpe0xb9q8qiLsFr0=vr0=vr0dc8meaabaqaciaacaGaaeqabaqabeGadaaakeaacuWGtbWugaacaaaa@2DEA@(**x**, **y**, A
 MathType@MTEF@5@5@+=feaafiart1ev1aaatCvAUfKttLearuWrP9MDH5MBPbIqV92AaeXatLxBI9gBamrtHrhAL1wy0L2yHvtyaeHbnfgDOvwBHrxAJfwnaebbnrfifHhDYfgasaacH8akY=wiFfYdH8Gipec8Eeeu0xXdbba9frFj0=OqFfea0dXdd9vqai=hGuQ8kuc9pgc9s8qqaq=dirpe0xb9q8qiLsFr0=vr0=vr0dc8meaabaqaciaacaGaaeqabaWaaeGaeaaakeaaimaacqWFaeFqaaa@3821@) of the local alignment of the two sequences is composed of a sum over all aligned pairs and a sum over all gaps of both sequences:

S˜(x,y,A)=∑k=1Kσ(xik,yjk)+∑gaps gg(lg)=∑k=1Kσ(xik,yjk)+∑k=1K−1{g(lgx(k))+g(lgy(k))}
 MathType@MTEF@5@5@+=feaafiart1ev1aaatCvAUfKttLearuWrP9MDH5MBPbIqV92AaeXatLxBI9gBaebbnrfifHhDYfgasaacH8akY=wiFfYdH8Gipec8Eeeu0xXdbba9frFj0=OqFfea0dXdd9vqai=hGuQ8kuc9pgc9s8qqaq=dirpe0xb9q8qiLsFr0=vr0=vr0dc8meaabaqaciaacaGaaeqabaqabeGadaaakeaafaqadeGabaaabaGafm4uamLbaGaacqGGOaakieqacqWF4baEcqGGSaalcqWF5bqEcqGGSaalt0uy0HwzTfgDPnwy1egaryqtHrhAL1wy0L2yHvdaiqaacqGFaeFqcqGGPaqkcqGH9aqpdaaeWbqaaGGaciab9n8aZjabcIcaOiabdIha4naaBaaaleaacqWGPbqAdaWgaaadbaGaem4AaSgabeaaaSqabaGccqGGSaalcqWG5bqEdaWgaaWcbaGaemOAaO2aaSbaaWqaaiabdUgaRbqabaaaleqaaOGaeiykaKIaey4kaSYaaabuaeaacqWGNbWzcqGGOaakcqWGSbaBdaWgaaWcbaGaem4zaCgabeaakiabcMcaPaWcbaGaee4zaCMaeeyyaeMaeeiCaaNaee4CamNaeeiiaaIaem4zaCgabeqdcqGHris5aaWcbaGaem4AaSMaeyypa0JaeGymaedabaGaem4saSeaniabggHiLdaakeaacqGH9aqpdaaeWbqaaiab9n8aZjabcIcaOiabdIha4naaBaaaleaacqWGPbqAdaWgaaadbaGaem4AaSgabeaaaSqabaGccqGGSaalcqWG5bqEdaWgaaWcbaGaemOAaO2aaSbaaWqaaiabdUgaRbqabaaaleqaaOGaeiykaKIaey4kaSYaaabCaeaacqGG7bWEcqWGNbWzcqGGOaakcqWGSbaBdaqhaaWcbaGaem4zaCgabaGaemiEaGhaaOGaeiikaGIaem4AaSMaeiykaKIaeiykaKIaey4kaSIaem4zaCMaeiikaGIaemiBaW2aa0baaSqaaiabdEgaNbqaaiabdMha5baakiabcIcaOiabdUgaRjabcMcaPiabcMcaPiabc2ha9bWcbaGaem4AaSMaeyypa0JaeGymaedabaGaem4saSKaeyOeI0IaeGymaedaniabggHiLdaaleaacqWGRbWAcqGH9aqpcqaIXaqmaeaacqWGlbWsa0GaeyyeIuoaaaaaaa@9D73@

where *σ *(*a*, *b*) *a, b *∈ A
 MathType@MTEF@5@5@+=feaafiart1ev1aaatCvAUfKttLearuWrP9MDH5MBPbIqV92AaeXatLxBI9gBamrtHrhAL1wy0L2yHvtyaeHbnfgDOvwBHrxAJfwnaebbnrfifHhDYfgasaacH8akY=wiFfYdH8Gipec8Eeeu0xXdbba9frFj0=OqFfea0dXdd9vqai=hGuQ8kuc9pgc9s8qqaq=dirpe0xb9q8qiLsFr0=vr0=vr0dc8meaabaqaciaacaGaaeqabaWaaeGaeaaakeaaimaacqWFaeFqaaa@3821@ is the given *score matrix *(or *substitution matrix*) and *g *(*l*) the *gap-cost function *with *g *(0) = 0. Note that the alignment is local, because the (possibly large) gaps at the beginning and the end of each sequence are not included in the scoring function. Otherwise the alignment would be global. Here, we consider the BLOSUM62 [20] and the PAM250 [21,22] matrices and affine gap costs, i.e. *g *(*l*) = *α *+ *β *(*l *-1). The *similarity *of the sequences is the optimum alignment with the maximum score

S(x,y)=max⁡AS˜(x,y,A),
 MathType@MTEF@5@5@+=feaafiart1ev1aaatCvAUfKttLearuWrP9MDH5MBPbIqV92AaeXatLxBI9gBaebbnrfifHhDYfgasaacH8akY=wiFfYdH8Gipec8Eeeu0xXdbba9frFj0=OqFfea0dXdd9vqai=hGuQ8kuc9pgc9s8qqaq=dirpe0xb9q8qiLsFr0=vr0=vr0dc8meaabaqaciaacaGaaeqabaqabeGadaaakeaacqWGtbWucqGGOaakieqacqWF4baEcqGGSaalcqWF5bqEcqGGPaqkcqGH9aqpdaWfqaqaaiGbc2gaTjabcggaHjabcIha4bWcbaWenfgDOvwBHrxAJfwnHbqeg0uy0HwzTfgDPnwy1aaceaGae4haXheabeaakiqbdofatzaaiaGaeiikaGIae8hEaGNaeiilaWIae8xEaKNaeiilaWIae4haXhKaeiykaKIaeiilaWcaaa@4E77@

which can be obtained in O
 MathType@MTEF@5@5@+=feaafiart1ev1aaatCvAUfKttLearuWrP9MDH5MBPbIqV92AaeXatLxBI9gBamrtHrhAL1wy0L2yHvtyaeHbnfgDOvwBHrxAJfwnaebbnrfifHhDYfgasaacH8akY=wiFfYdH8Gipec8Eeeu0xXdbba9frFj0=OqFfea0dXdd9vqai=hGuQ8kuc9pgc9s8qqaq=dirpe0xb9q8qiLsFr0=vr0=vr0dc8meaabaqaciaacaGaaeqabaWaaeGaeaaakeaaimaacqWFoe=taaa@383D@(*LM*) time [7].

In the case of gapless optimum local alignments of two random sequences of *L *and *M *independent letters from Σ with frequencies {*f*_*a *_} with *a *∈ Σ and ∑_*a *_*f*_*a *_= 1, referred as *null model*, the score statistics can be calculated analytically in the asymptotic regime of long sequences [9,10].

In this case one obtains the Gumbel distribution (Karlin-Altschul statistics) [23]

ℙ(*S *≥ *b*) = 1 - exp [- *KLM e*^-*λb*^]

or

P_Gumble _(*s*) = ℙ(*S *= *s*) = *λ KLM *exp [-*λ s *- *KLM e*^-*λ s*^]

The parameters *λ *and *K *of Eq. (3) can be derived directly from the score matrix *σ *(*a*, *b*) and frequencies *f*_*a *_[9,10].

As pointed out by Altschul and Gish [16], in finite systems there occur edge effects: An alignment may extend to the end of either sequence and the score will be distorted towards lower values and high scores become less probable. Since this effect vanishes in the limit of infinite sequences, the tail of Eq. (3) can be understood as an upper bound for finite sequences.

Arratia and Waterman [24] predicted a phase transition between a linear phase and a logarithmic phase, i.e. a linear growth of the excepted score as a function of the sequence length, changing to a logarithmic growth with increasing gap costs. In the linear phase an optimum alignment may spread over a large range of the sequences and the statistical theory breaks down. However, only the logarithmic phase is of interest in biological questions because the alignment algorithm becomes more sensitive in this phase, especially near the threshold [25].

Often the sensitivity of an alignment algorithm can be increased by not only considering the best optimal alignment score, but also the *k*-best scores of non overlapping alignments. An O
 MathType@MTEF@5@5@+=feaafiart1ev1aaatCvAUfKttLearuWrP9MDH5MBPbIqV92AaeXatLxBI9gBamrtHrhAL1wy0L2yHvtyaeHbnfgDOvwBHrxAJfwnaebbnrfifHhDYfgasaacH8akY=wiFfYdH8Gipec8Eeeu0xXdbba9frFj0=OqFfea0dXdd9vqai=hGuQ8kuc9pgc9s8qqaq=dirpe0xb9q8qiLsFr0=vr0=vr0dc8meaabaqaciaacaGaaeqabaWaaeGaeaaakeaaimaacqWFoe=taaa@383D@(*LM*) algorithm for this task, based on Sellers concept of local optimality, was developed [26,27]. According to Karlin and Altschul [28] also the sum statistics of the *k*-best alignment scores for random sequences can be derived analytically for asymptotically long sequences. The probability *f *for the sum of the *k*-best normalized scores Tk=λ∑ik(Si−ln⁡KLMλ)
 MathType@MTEF@5@5@+=feaafiart1ev1aaatCvAUfKttLearuWrP9MDH5MBPbIqV92AaeXatLxBI9gBaebbnrfifHhDYfgasaacH8akY=wiFfYdH8Gipec8Eeeu0xXdbba9frFj0=OqFfea0dXdd9vqai=hGuQ8kuc9pgc9s8qqaq=dirpe0xb9q8qiLsFr0=vr0=vr0dc8meaabaqaciaacaGaaeqabaqabeGadaaakeaacqWGubavdaWgaaWcbaGaem4AaSgabeaakiabg2da9GGaciab=T7aSnaaqadabaGaeiikaGIaem4uam1aaSbaaSqaaiabdMgaPbqabaGccqGHsisldaWcaaqaaiGbcYgaSjabc6gaUjabdUealjabdYeamjabd2eanbqaaiab=T7aSbaaaSqaaiabdMgaPbqaaiabdUgaRbqdcqGHris5aOGaeiykaKcaaa@4440@ (*λ *and *K *are the corresponding Gumbel-parameters for the optimal alignment)is given by the integral

f(t)=e−tk!(k−2)!∫0∞yk−2exp⁡(−e(y−t)/k)dy.
 MathType@MTEF@5@5@+=feaafiart1ev1aaatCvAUfKttLearuWrP9MDH5MBPbIqV92AaeXatLxBI9gBaebbnrfifHhDYfgasaacH8akY=wiFfYdH8Gipec8Eeeu0xXdbba9frFj0=OqFfea0dXdd9vqai=hGuQ8kuc9pgc9s8qqaq=dirpe0xb9q8qiLsFr0=vr0=vr0dc8meaabaqaciaacaGaaeqabaqabeGadaaakeaacqWGMbGzcqGGOaakcqWG0baDcqGGPaqkcqGH9aqpdaWcaaqaaiabdwgaLnaaCaaaleqabaGaeyOeI0IaemiDaqhaaaGcbaGaem4AaSMaeiyiaeIaeiikaGIaem4AaSMaeyOeI0IaeGOmaiJaeiykaKIaeiyiaecaamaapedabaGaemyEaK3aaWbaaSqabeaacqWGRbWAcqGHsislcqaIYaGmaaGccyGGLbqzcqGG4baEcqGGWbaCcqGGOaakcqGHsislcqWGLbqzdaahaaWcbeqaaiabcIcaOiabdMha5jabgkHiTiabdsha0jabcMcaPiabc+caViabdUgaRbaakiabcMcaPiabdsgaKjabdMha5jabc6caUaWcbaGaeGimaadabaGaeyOhIukaniabgUIiYdaaaa@5B5A@

In the tail, i.e. for large *t*, *f *(*t*) is well approximated by

ftail(t)=e−tk!(k−1)![tk−1−(k−1)tk−2)].
 MathType@MTEF@5@5@+=feaafiart1ev1aaatCvAUfKttLearuWrP9MDH5MBPbIqV92AaeXatLxBI9gBaebbnrfifHhDYfgasaacH8akY=wiFfYdH8Gipec8Eeeu0xXdbba9frFj0=OqFfea0dXdd9vqai=hGuQ8kuc9pgc9s8qqaq=dirpe0xb9q8qiLsFr0=vr0=vr0dc8meaabaqaciaacaGaaeqabaqabeGadaaakeaacqWGMbGzdaWgaaWcbaGaeeiDaqNaeeyyaeMaeeyAaKMaeeiBaWgabeaakiabcIcaOiabdsha0jabcMcaPiabg2da9maalaaabaGaemyzau2aaWbaaSqabeaacqGHsislcqWG0baDaaaakeaacqWGRbWAcqGGHaqicqGGOaakcqWGRbWAcqGHsislcqaIXaqmcqGGPaqkcqGGHaqiaaGaei4waSLaemiDaq3aaWbaaSqabeaacqWGRbWAcqGHsislcqaIXaqmaaGccqGHsislcqGGOaakcqWGRbWAcqGHsislcqaIXaqmcqGGPaqkcqWG0baDdaahaaWcbeqaaiabdUgaRjabgkHiTiabikdaYaaakiabcMcaPiabc2faDjabc6caUaaa@578C@

In the asymptotic theory the score can be seen as a continuous variable and the probabilities Eq. (4) and Eq. (5) become probability densities. Then the probability of finding a normalized score *b *or larger is given by the integral ℙ(S≥b)=∫b∞f(t)dt
 MathType@MTEF@5@5@+=feaafiart1ev1aaatCvAUfKttLearuWrP9MDH5MBPbIqV92AaeXatLxBI9gBaebbnrfifHhDYfgasaacH8akY=wiFfYdH8Gipec8Eeeu0xXdbba9frFj0=OqFfea0dXdd9vqai=hGuQ8kuc9pgc9s8qqaq=dirpe0xb9q8qiLsFr0=vr0=vr0dc8meaabaqaciaacaGaaeqabaqabeGadaaakeaatuuDJXwAK1uy0HMmaeHbfv3ySLgzG0uy0HgiuD3BaGabaiab=LriqjabcIcaOiabdofatjabgwMiZkabdkgaIjabcMcaPiabg2da9maapedabaGaemOzayMaeiikaGIaemiDaqNaeiykaKIaemizaqMaemiDaqhaleaacqWGIbGyaeaacqGHEisPa0Gaey4kIipaaaa@4A78@. However in computer simulations the score is a discrete variable and therefore the normalization constants in Eq. (5) differ from continious scoring. Below we will compare the results of our numerical studies to this distribution in the tail of the data for values *k *= 2, ..., 5.

## Sampling of rare-events

### Metropolis Hastings Algorithm

As already pointed out, the main purpose of this paper is to calculate the tail of the distribution of optimum scores of gapped local alignments over pairs of randomly and independently drawn sequences of finite lengths. The basic idea of our approach is to generate the sequences from different distributions, which are biased towards higher scores.

In order to be more precise let us denote the state space of all possible pairs of sequences (**x**, **y**) as X
 MathType@MTEF@5@5@+=feaafiart1ev1aaatCvAUfKttLearuWrP9MDH5MBPbIqV92AaeXatLxBI9gBamrtHrhAL1wy0L2yHvtyaeHbnfgDOvwBHrxAJfwnaebbnrfifHhDYfgasaacH8akY=wiFfYdH8Gipec8Eeeu0xXdbba9frFj0=OqFfea0dXdd9vqai=hGuQ8kuc9pgc9s8qqaq=dirpe0xb9q8qiLsFr0=vr0=vr0dc8meaabaqaciaacaGaaeqabaWaaeGaeaaakeaaimaacqWFxepwaaa@384F@ and an element in this space as a *configuration*. We write **X **= (**x**, **y**).

The probability mass function (pmf) of finding **X **under the null model is given by p(X)=p(x,y)=∏i=1Lfxi∏j=1Mfyj
MathType@MTEF@5@5@+=feaafiart1ev1aaatCvAUfKttLearuWrP9MDH5MBPbIqV92AaeXatLxBI9gBaebbnrfifHhDYfgasaacH8akY=wiFfYdH8Gipec8Eeeu0xXdbba9frFj0=OqFfea0dXdd9vqai=hGuQ8kuc9pgc9s8qqaq=dirpe0xb9q8qiLsFr0=vr0=vr0dc8meaabaqaciaacaGaaeqabaqabeGadaaakeaacqWGWbaCcqGGOaakieqacqWFybawcqGGPaqkcqGH9aqpcqWGWbaCcqGGOaakcqWF4baEcqGGSaalcqWF5bqEcqGGPaqkcqGH9aqpdaqeWaqaaiabdAgaMnaaBaaaleaacqWG4baEdaWgaaadbaGaemyAaKgabeaaaSqabaaabaGaemyAaKMaeyypa0JaeGymaedabaGaemitaWeaniabg+GivdGcdaqeWaqaaiabdAgaMnaaBaaaleaacqWG5bqEdaWgaaadbaGaemOAaOgabeaaaSqabaaabaGaemOAaOMaeyypa0JaeGymaedabaGaemyta0eaniabg+Givdaaaa@4FD5@ and the alignment score as defined in Eq. (2) is a random variable. A direct way to obtain the probability of the occurrence of a certain score *s*, is to generate *n *uncorrelated representatives **X**_*i *_∈ X
 MathType@MTEF@5@5@+=feaafiart1ev1aaatCvAUfKttLearuWrP9MDH5MBPbIqV92AaeXatLxBI9gBamrtHrhAL1wy0L2yHvtyaeHbnfgDOvwBHrxAJfwnaebbnrfifHhDYfgasaacH8akY=wiFfYdH8Gipec8Eeeu0xXdbba9frFj0=OqFfea0dXdd9vqai=hGuQ8kuc9pgc9s8qqaq=dirpe0xb9q8qiLsFr0=vr0=vr0dc8meaabaqaciaacaGaaeqabaWaaeGaeaaakeaaimaacqWFxepwaaa@384F@ according to the null model and then compute the expectation values of the family of indicator functions *h*_*s*_: X
 MathType@MTEF@5@5@+=feaafiart1ev1aaatCvAUfKttLearuWrP9MDH5MBPbIqV92AaeXatLxBI9gBamrtHrhAL1wy0L2yHvtyaeHbnfgDOvwBHrxAJfwnaebbnrfifHhDYfgasaacH8akY=wiFfYdH8Gipec8Eeeu0xXdbba9frFj0=OqFfea0dXdd9vqai=hGuQ8kuc9pgc9s8qqaq=dirpe0xb9q8qiLsFr0=vr0=vr0dc8meaabaqaciaacaGaaeqabaWaaeGaeaaakeaaimaacqWFxepwaaa@384F@ → ℝ with *h*_*s *_(**X**) = 1, if *S *(**X**) = *s *and *h*_*s *_(**X**) = 0 otherwise, in other words

ℙ[S(X)=s]=E[hs(X)]=∑Xhs(X)p(X)≈1n∑i=1nhs(Xi).
 MathType@MTEF@5@5@+=feaafiart1ev1aaatCvAUfKttLearuWrP9MDH5MBPbIqV92AaeXatLxBI9gBaebbnrfifHhDYfgasaacH8akY=wiFfYdH8Gipec8Eeeu0xXdbba9frFj0=OqFfea0dXdd9vqai=hGuQ8kuc9pgc9s8qqaq=dirpe0xb9q8qiLsFr0=vr0=vr0dc8meaabaqaciaacaGaaeqabaqabeGadaaakeaatuuDJXwAK1uy0HMmaeHbfv3ySLgzG0uy0HgiuD3BaGabaiab=LriqjabcUfaBjabdofatjabcIcaOGqabiab+HfayjabcMcaPiabg2da9iabdohaZjabc2faDjabg2da9iab=ri8fjabcUfaBjabdIgaOnaaBaaaleaacqWGZbWCaeqaaOGaeiikaGIae4hwaGLaeiykaKIaeiyxa0Laeyypa0ZaaabuaeaacqWGObaAdaWgaaWcbaGaem4CamhabeaaaeaacqGFybawaeqaniabggHiLdGccqGGOaakcqGFybawcqGGPaqkcqWGWbaCcqGGOaakcqGFybawcqGGPaqkcqGHijYUdaWcaaqaaiabigdaXaqaaiabd6gaUbaadaaeWbqaaiabdIgaOnaaBaaaleaacqWGZbWCaeqaaOGaeiikaGIae4hwaG1aaSbaaSqaaiabdMgaPbqabaGccqGGPaqkaSqaaiabdMgaPjabg2da9iabigdaXaqaaiabd6gaUbqdcqGHris5aOGaeiOla4caaa@6E0F@

Since the region of biological interest is located in the rare-event tail a huge amount of samples would be needed to achieve an acceptable accuracy. In practice the rare-event tail becomes inaccessible.

Our method is based on importance sampling of a mixture of chains based on the Metropolis-Hastings algorithm. Before describing the coupling of multiple chains, we introduce the general idea of importance sampling first: The approach is based on sampling from a different distribution, such that the region of interest is sampled with high probability. Since this happens in a controlled manner the true distribution can be obtained afterward, as frequently used in variance reduction techniques. The modified distribution yields a different random variable with a different pmf *q*. We may write

P(s)=ℙ[S(X)=s]=∑X′hs(X′)p(X′)q(X′)q(X′)≈1n∑i=1nhs(X′i)p(X′i)q(X′i).
 MathType@MTEF@5@5@+=feaafiart1ev1aaatCvAUfKttLearuWrP9MDH5MBPbIqV92AaeXatLxBI9gBaebbnrfifHhDYfgasaacH8akY=wiFfYdH8Gipec8Eeeu0xXdbba9frFj0=OqFfea0dXdd9vqai=hGuQ8kuc9pgc9s8qqaq=dirpe0xb9q8qiLsFr0=vr0=vr0dc8meaabaqaciaacaGaaeqabaqabeGadaaakeaacqWGqbaucqGGOaakcqWGZbWCcqGGPaqkcqGH9aqptuuDJXwAK1uy0HMmaeHbfv3ySLgzG0uy0HgiuD3BaGabaiab=LriqjabcUfaBjabdofatjabcIcaOGqabiab+HfayjabcMcaPiabg2da9iabdohaZjabc2faDjabg2da9maaqafabaGaemiAaG2aaSbaaSqaaiabdohaZbqabaGccqGGOaakcuGFybawgaqbaiabcMcaPmaalaaabaGaemiCaaNaeiikaGIaf4hwaGLbauaacqGGPaqkaeaacqWGXbqCcqGGOaakcuGFybawgaqbaiabcMcaPaaaaSqaaiqb+HfayzaafaaabeqdcqGHris5aOGaemyCaeNaeiikaGIaf4hwaGLbauaacqGGPaqkcqGHijYUdaWcaaqaaiabigdaXaqaaiabd6gaUbaadaaeWbqaaiabdIgaOnaaBaaaleaacqWGZbWCaeqaaaqaaiabdMgaPjabg2da9iabigdaXaqaaiabd6gaUbqdcqGHris5aOGaeiikaGIaf4hwaGLbauaadaWgaaWcbaGaemyAaKgabeaakiabcMcaPmaalaaabaGaemiCaaNaeiikaGIaf4hwaGLbauaadaWgaaWcbaGaemyAaKgabeaakiabcMcaPaqaaiabdghaXjabcIcaOiqb+HfayzaafaWaaSbaaSqaaiabdMgaPbqabaGccqGGPaqkaaGaeiOla4caaa@7C9B@

At least approximately, the distribution of local alignment follows a Gumbel distribution, which exhibits an exponential behavior in the tail. Therefore an obvious choice for the biased distribution is

qT(X)≡q˜T(X)ZT≡1ZTp(X)⋅exp⁡[S(X)/T],
 MathType@MTEF@5@5@+=feaafiart1ev1aaatCvAUfKttLearuWrP9MDH5MBPbIqV92AaeXatLxBI9gBaebbnrfifHhDYfgasaacH8akY=wiFfYdH8Gipec8Eeeu0xXdbba9frFj0=OqFfea0dXdd9vqai=hGuQ8kuc9pgc9s8qqaq=dirpe0xb9q8qiLsFr0=vr0=vr0dc8meaabaqaciaacaGaaeqabaqabeGadaaakeaacqWGXbqCdaWgaaWcbaGaemivaqfabeaakiabcIcaOGqabiab=HfayjabcMcaPiabggMi6oaalaaabaGafmyCaeNbaGaadaWgaaWcbaGaemivaqfabeaakiabcIcaOiabdIfayjabcMcaPaqaaiabdQfaAnaaBaaaleaacqWGubavaeqaaaaakiabggMi6oaalaaabaGaeGymaedabaGaemOwaO1aaSbaaSqaaiabdsfaubqabaaaaOGaemiCaaNaeiikaGIae8hwaGLaeiykaKIaeyyXICTagiyzauMaeiiEaGNaeiiCaaNaei4waSLaem4uamLaeiikaGIae8hwaGLaeiykaKIaei4la8IaemivaqLaeiyxa0LaeiilaWcaaa@5680@

where q˜T
 MathType@MTEF@5@5@+=feaafiart1ev1aaatCvAUfKttLearuWrP9MDH5MBPbIqV92AaeXatLxBI9gBaebbnrfifHhDYfgasaacH8akY=wiFfYdH8Gipec8Eeeu0xXdbba9frFj0=OqFfea0dXdd9vqai=hGuQ8kuc9pgc9s8qqaq=dirpe0xb9q8qiLsFr0=vr0=vr0dc8meaabaqaciaacaGaaeqabaqabeGadaaakeaacuWGXbqCgaacamaaBaaaleaacqWGubavaeqaaaaa@2F83@ the unnormalized weight of a configuration, *Z*_*T *_is a (usually unknown) normalization constant and *T *an adjustable parameter, which we will call "temperature" (In the framework of statistical mechanics, which is closely related to our method, the parameter *T *describes the temperature of a physical system. The pair of sequences can be seen as a configuration of a physical system and the negative score as the energy function. Then exp [*S *(**X**)/*T*] refers to the so called *Gibbs-Boltzmann distribution*.) The close-to Gumbel form of the distribution is also directly related to the so called "large deviation rate function", which basically describes the decay rate of the tail of the distribution. Note that, if the score distribution is an exact Gumbel distribution Eq. (3), i.e. the rate function a known constant *λ*, then setting *T *= 1/*λ *in Eq. (7) yields a "flat score histogram" for sufficient large *s*. Hence, in this case, a simulation at a single carfully chosen value *T *would be sufficient to obtain the full result. Since *P *(*s*) does not follow the Gumbel form exactly, importance sampling has to be applied. Each value of *T *selects one region of the distribution around which a high accurracy is obtained.

This importance sampling approach is conceptual related to the method of "measure change" in large deviation theory. For example Siegmund and Yakir [14] approximated the p-value for local sequence alignment by considering the log-likelihood ratio between an alternative measure and the measure of the null model. Under the new measure a rare event occurs more likely than under the original null measure and approximations become possible. Another example can be found in Ref. [29], where techniques from large deviation theory were applied to proof "asymptotic efficiency" of rare-event simulations.

However, since there is no direct method to sample directly according to the modified distribution Eq. (7) we implemented the *Metropolis-Hastings algorithm *[30], which is explained now in detail. It is based on ergodic *Markov chain Monte Carlo (MCMC) *in state space. Ergodic here means, that for a given state in the configuration space X
 MathType@MTEF@5@5@+=feaafiart1ev1aaatCvAUfKttLearuWrP9MDH5MBPbIqV92AaeXatLxBI9gBamrtHrhAL1wy0L2yHvtyaeHbnfgDOvwBHrxAJfwnaebbnrfifHhDYfgasaacH8akY=wiFfYdH8Gipec8Eeeu0xXdbba9frFj0=OqFfea0dXdd9vqai=hGuQ8kuc9pgc9s8qqaq=dirpe0xb9q8qiLsFr0=vr0=vr0dc8meaabaqaciaacaGaaeqabaWaaeGaeaaakeaaimaacqWFxepwaaa@384F@ any other can be achieved by stepwise "local" modifications of configurations in finite time. Note that we work in discrete time steps here. Let **X **∈ X
 MathType@MTEF@5@5@+=feaafiart1ev1aaatCvAUfKttLearuWrP9MDH5MBPbIqV92AaeXatLxBI9gBamrtHrhAL1wy0L2yHvtyaeHbnfgDOvwBHrxAJfwnaebbnrfifHhDYfgasaacH8akY=wiFfYdH8Gipec8Eeeu0xXdbba9frFj0=OqFfea0dXdd9vqai=hGuQ8kuc9pgc9s8qqaq=dirpe0xb9q8qiLsFr0=vr0=vr0dc8meaabaqaciaacaGaaeqabaWaaeGaeaaakeaaimaacqWFxepwaaa@384F@ a configuration at time *t *(e.g. at the start of the simulation). To determine the configuration at time *t *+ 1, first a *trial configuration *X* is selected randomly among its "neighbors". The neighborhood of a configuration depends on the choice of trial steps, which are specified below. For practical reasons we require, that the score within a neighborhood of a given configuration will not change too much. The transition matrix for this trial selection process is denoted by *P *(**X**, X*). Now, the trial configuration becomes the configuration at time *t *+ 1, i.e. is *accepted*, with probability

p˜(X→X∗)=max⁡{1,P(X∗,X)P(X,X∗)⋅qT(X∗)qT(X)}=max⁡{1,P(X∗,X)P(X,X∗)exp⁡[ΔS/T]},
 MathType@MTEF@5@5@+=feaafiart1ev1aaatCvAUfKttLearuWrP9MDH5MBPbIqV92AaeXatLxBI9gBaebbnrfifHhDYfgasaacH8akY=wiFfYdH8Gipec8Eeeu0xXdbba9frFj0=OqFfea0dXdd9vqai=hGuQ8kuc9pgc9s8qqaq=dirpe0xb9q8qiLsFr0=vr0=vr0dc8meaabaqaciaacaGaaeqabaqabeGadaaakeaacuWGWbaCgaacaiabcIcaOGqabiab=HfayjabgkziUkab=HfaynaaCaaaleqabaGaey4fIOcaaOGaeiykaKIaeyypa0JagiyBa0MaeiyyaeMaeiiEaG3aaiWabeaacqaIXaqmcqGGSaaldaWcaaqaaiabdcfaqjabcIcaOiab=HfaynaaCaaaleqabaGaey4fIOcaaOGaeiilaWIae8hwaGLaeiykaKcabaGaemiuaaLaeiikaGIae8hwaGLaeiilaWIae8hwaG1aaWbaaSqabeaacqGHxiIkaaGccqGGPaqkaaGaeyyXIC9aaSaaaeaacqWGXbqCdaWgaaWcbaGaemivaqfabeaakiabcIcaOiab=HfaynaaCaaaleqabaGaey4fIOcaaOGaeiykaKcabaGaemyCae3aaSbaaSqaaiabdsfaubqabaGccqGGOaakcqWFybawcqGGPaqkaaaacaGL7bGaayzFaaGaeyypa0JagiyBa0MaeiyyaeMaeiiEaG3aaiWabeaacqaIXaqmcqGGSaaldaWcaaqaaiabdcfaqjabcIcaOiab=HfaynaaCaaaleqabaGaey4fIOcaaOGaeiilaWIae8hwaGLaeiykaKcabaGaemiuaaLaeiikaGIae8hwaGLaeiilaWIae8hwaG1aaWbaaSqabeaacqGHxiIkaaGccqGGPaqkaaGagiyzauMaeiiEaGNaeiiCaaNaei4waSLaeuiLdqKaem4uamLaei4la8IaemivaqLaeiyxa0facaGL7bGaayzFaaGaeiilaWcaaa@8037@

with Δ*S *= *S *(X*) - *S *(**X**) If the trial configuration is not accepted, the previous configuration **X **is kept for the next time step *t *+ 1. In this way, the Markov chain fulfills the detailed balance condition *P *(X*, **X**)p˜
 MathType@MTEF@5@5@+=feaafiart1ev1aaatCvAUfKttLearuWrP9MDH5MBPbIqV92AaeXatLxBI9gBaebbnrfifHhDYfgasaacH8akY=wiFfYdH8Gipec8Eeeu0xXdbba9frFj0=OqFfea0dXdd9vqai=hGuQ8kuc9pgc9s8qqaq=dirpe0xb9q8qiLsFr0=vr0=vr0dc8meaabaqaciaacaGaaeqabaqabeGadaaakeaacuWGWbaCgaacaaaa@2E24@(X* → **X**)·*q*_*T *_(X*) = *P *(**X**, X*)p˜
 MathType@MTEF@5@5@+=feaafiart1ev1aaatCvAUfKttLearuWrP9MDH5MBPbIqV92AaeXatLxBI9gBaebbnrfifHhDYfgasaacH8akY=wiFfYdH8Gipec8Eeeu0xXdbba9frFj0=OqFfea0dXdd9vqai=hGuQ8kuc9pgc9s8qqaq=dirpe0xb9q8qiLsFr0=vr0=vr0dc8meaabaqaciaacaGaaeqabaqabeGadaaakeaacuWGWbaCgaacaaaa@2E24@(**X **→ X*)·*q*_*T*_(**X**). In this case it has been proven that an ergodic Markov chain converges to the stationary distribution *q*_*T*_. Ergodicity means, that there is a non-zero probability for a path between *any *pair(**X**_**1**_, **X**_**2**_) of configurations.

We used a simple way to define the neighborhood of a configuration and constructed the trial configuration as follows: First a letter *a *is drawn from the alphabet Σ according to the letter weights *f*_*a *_and next one of the sequences (**x **or **y**) and a position *i *is chosen randomly. Finally, the letter at position *i *is replaced by *a*.

Given a Monte Carlo chain (**X**_1_, ..., **X**_*n*_) estimated for a fixed temperature *T *in principle one may estimate expectation values with respect to any member of the family of distributions *q*_*T *_by importance reweighting

ET′[g(X)]≈1n∑i=1nqT′(Xi)qT(Xi)⋅g(Xi)
 MathType@MTEF@5@5@+=feaafiart1ev1aaatCvAUfKttLearuWrP9MDH5MBPbIqV92AaeXatLxBI9gBaebbnrfifHhDYfgasaacH8akY=wiFfYdH8Gipec8Eeeu0xXdbba9frFj0=OqFfea0dXdd9vqai=hGuQ8kuc9pgc9s8qqaq=dirpe0xb9q8qiLsFr0=vr0=vr0dc8meaabaqaciaacaGaaeqabaqabeGadaaakeaatuuDJXwAK1uy0HMmaeHbfv3ySLgzG0uy0HgiuD3BaGabaiab=ri8fnaaBaaaleaacuWGubavgaqbaaqabaGccqGGBbWwcqWGNbWzcqGGOaakieqacqGFybawcqGGPaqkcqGGDbqxcqGHijYUdaWcaaqaaiabigdaXaqaaiabd6gaUbaadaaeWbqaamaalaaabaGaemyCae3aaSbaaSqaaiqbdsfauzaafaaabeaakiabcIcaOiab+HfaynaaBaaaleaacqWGPbqAaeqaaOGaeiykaKcabaGaemyCae3aaSbaaSqaaiabdsfaubqabaGccqGGOaakcqGFybawdaWgaaWcbaGaemyAaKgabeaakiabcMcaPaaaaSqaaiabdMgaPjabg2da9iabigdaXaqaaiabd6gaUbqdcqGHris5aOGaeyyXICTaem4zaCMaeiikaGIae4hwaG1aaSbaaSqaaiabdMgaPbqabaGccqGGPaqkaaa@62A4@

Since the normalization of *q*_*T *_is not trivial, we used a different normalization

ET′[g(X)]≈1n∑i=1nq˜T′(Xi)q˜T(Xi)⋅g(Xi),
 MathType@MTEF@5@5@+=feaafiart1ev1aaatCvAUfKttLearuWrP9MDH5MBPbIqV92AaeXatLxBI9gBaebbnrfifHhDYfgasaacH8akY=wiFfYdH8Gipec8Eeeu0xXdbba9frFj0=OqFfea0dXdd9vqai=hGuQ8kuc9pgc9s8qqaq=dirpe0xb9q8qiLsFr0=vr0=vr0dc8meaabaqaciaacaGaaeqabaqabeGadaaakeaatuuDJXwAK1uy0HMmaeHbfv3ySLgzG0uy0HgiuD3BaGabaiab=ri8fnaaBaaaleaacuWGubavgaqbaaqabaGccqGGBbWwcqWGNbWzcqGGOaakieqacqGFybawcqGGPaqkcqGGDbqxcqGHijYUdaWcaaqaaiabigdaXaqaaiabd6gaUbaadaaeWbqaamaalaaabaGafmyCaeNbaGaadaWgaaWcbaGafmivaqLbauaaaeqaaOGaeiikaGIae4hwaG1aaSbaaSqaaiabdMgaPbqabaGccqGGPaqkaeaacuWGXbqCgaacamaaBaaaleaacqWGubavaeqaaOGaeiikaGIae4hwaG1aaSbaaSqaaiabdMgaPbqabaGccqGGPaqkaaaaleaacqWGPbqAcqGH9aqpcqaIXaqmaeaacqWGUbGBa0GaeyyeIuoakiabgwSixlabdEgaNjabcIcaOiab+HfaynaaBaaaleaacqWGPbqAaeqaaOGaeiykaKIaeiilaWcaaa@63A2@

and estimate *Z *from the sample Z=∑k=1nq˜T′(Xk)/q˜T(Xk)
 MathType@MTEF@5@5@+=feaafiart1ev1aaatCvAUfKttLearuWrP9MDH5MBPbIqV92AaeXatLxBI9gBaebbnrfifHhDYfgasaacH8akY=wiFfYdH8Gipec8Eeeu0xXdbba9frFj0=OqFfea0dXdd9vqai=hGuQ8kuc9pgc9s8qqaq=dirpe0xb9q8qiLsFr0=vr0=vr0dc8meaabaqaciaacaGaaeqabaqabeGadaaakeaacqWGAbGwcqGH9aqpdaaeWaqaaiqbdghaXzaaiaWaaSbaaSqaaiqbdsfauzaafaaabeaaaeaacqWGRbWAcqGH9aqpcqaIXaqmaeaacqWGUbGBa0GaeyyeIuoakiabcIcaOGqabiab=HfaynaaBaaaleaacqWGRbWAaeqaaOGaeiykaKIaei4la8IafmyCaeNbaGaadaWgaaWcbaGaemivaqfabeaakiabcIcaOiab=HfaynaaBaaaleaacqWGRbWAaeqaaOGaeiykaKcaaa@4556@. A detailed discussion about this issue can be found in Ref. [31,32]. In practice this may work badly as soon as the parameter ranges of the given distribution and the target distribution do not overlap sufficiently. In this case *q*_*T'*_(**X**_*i*_) is very small, but the configurations where *q*_*T' *_(**X**)/*q*_*T *_(**X**) is sufficiently large are not generated because *q*_*T *_(**X**) is relatively small for those. Therefore we sampled a mixture of many coupled Monte Carlo chains and reweighted the mixture, which is explained in detail in the next section. This allows for large overlap between neighboring distributions and to determine the normalization constants, up to an irrelevant global constant.

### Metropolis Coupled MCMC

*Metropolis Coupled Markov Chain Monte Carlo (MCMCMC) *was first invented by Charles Geyer [33] and then reinvented by Hukushima and Nemoto [34] under the term *exchange Monte Carlo*. In physical literature MCMCMC is often denoted as *parallel tempering*. The method has become a standard tool in disordered systems with a rough (free) energy landscape [35]. These rough energy landscapes are characterized by high energy barriers and can be found for problems like protein folding [36-40], nucleation [41], spin-glasses [42,43] and other models characterized by rare events [19,44]. In the last decade it turned out that MCMCMC accelerates equilibration and mixing remarkably.

In the framework of MCMCMC *m *copies **X**^(1)^, ..., **X**^(*m*) ^of the system held at different temperatures *T*_1 _<*T*_2 _< ... <*T*_*m *_are simulated in parallel. This means one samples from the product of the state space X
 MathType@MTEF@5@5@+=feaafiart1ev1aaatCvAUfKttLearuWrP9MDH5MBPbIqV92AaeXatLxBI9gBamrtHrhAL1wy0L2yHvtyaeHbnfgDOvwBHrxAJfwnaebbnrfifHhDYfgasaacH8akY=wiFfYdH8Gipec8Eeeu0xXdbba9frFj0=OqFfea0dXdd9vqai=hGuQ8kuc9pgc9s8qqaq=dirpe0xb9q8qiLsFr0=vr0=vr0dc8meaabaqaciaacaGaaeqabaWaaeGaeaaakeaaimaacqWFxepwaaa@384F@^*m *^weighted with the joint distribution with weights ∏j=1mqTj
MathType@MTEF@5@5@+=feaafiart1ev1aaatCvAUfKttLearuWrP9MDH5MBPbIqV92AaeXatLxBI9gBaebbnrfifHhDYfgasaacH8akY=wiFfYdH8Gipec8Eeeu0xXdbba9frFj0=OqFfea0dXdd9vqai=hGuQ8kuc9pgc9s8qqaq=dirpe0xb9q8qiLsFr0=vr0=vr0dc8meaabaqaciaacaGaaeqabaqabeGadaaakeaadaqeWaqaaiabdghaXnaaBaaaleaacqWGubavdaWgaaadbaGaemOAaOgabeaaaSqabaaabaGaemOAaOMaeyypa0JaeGymaedabaGaemyBa0ganiabg+Givdaaaa@37A5@. Since the different copies are allowed to exchange temperatures during the simulation, let us define the space of all possible mappings from the *m *configurations to the *m *temperatures as *temperature space*.

During the simulation, mainly each of the replicated configurations will evolve independently according the underlying MCMC scheme charaterized by the weight Eq. (7) at its current temperature, i.e. according to Eq. (8). In addition to this evolution, every *t*_exchange_*th *step (for each replicated configuration) a flip between two neighboring replicas *k *and *k *+ 1 is attempted, i.e. for all *k *∈ {1, ..., *m *- 1}. If an attempt is successful, the configurations **X**^(*k*) ^and **X**^(*k*+1) ^are exchanged (denoted by **X**^(*k*) ^↔ **X**^(*k*+1)^), i.e. the configurations which has previously evolved at temperature *T*_*k *_will now evolve at temperature *T*_*k *+ 1 _and vice versa. This exchange is accepted with the probability

p˜(X(k)↔X(k+1))=max⁡{1,qTk(X(k+1))qTk(X(k))⋅qTk+1(X(k))qTk+1(X(k+1))}=max⁡{1,exp⁡[−ΔβkΔS]},
 MathType@MTEF@5@5@+=feaafiart1ev1aaatCvAUfKttLearuWrP9MDH5MBPbIqV92AaeXatLxBI9gBaebbnrfifHhDYfgasaacH8akY=wiFfYdH8Gipec8Eeeu0xXdbba9frFj0=OqFfea0dXdd9vqai=hGuQ8kuc9pgc9s8qqaq=dirpe0xb9q8qiLsFr0=vr0=vr0dc8meaabaqaciaacaGaaeqabaqabeGadaaakeaafaqadeGabaaabaGafmiCaaNbaGaadaqadaqaaGqabiab=HfaynaaCaaaleqabaGaeiikaGIaem4AaSMaeiykaKcaaOGaeyiLHSQae8hwaG1aaWbaaSqabeaacqGGOaakcqWGRbWAcqGHRaWkcqaIXaqmcqGGPaqkaaaakiaawIcacaGLPaaacqGH9aqpcyGGTbqBcqGGHbqycqGG4baEdaGadeqaaiabigdaXiabcYcaSmaalaaabaGaemyCae3aaSbaaSqaaiabdsfaunaaBaaameaacqWGRbWAaeqaaaWcbeaakiabcIcaOiab=HfaynaaCaaaleqabaGaeiikaGIaem4AaSMaey4kaSIaeGymaeJaeiykaKcaaOGaeiykaKcabaGaemyCae3aaSbaaSqaaiabdsfaunaaBaaameaacqWGRbWAaeqaaaWcbeaakiabcIcaOiab=HfaynaaCaaaleqabaGaeiikaGIaem4AaSMaeiykaKcaaOGaeiykaKcaaiabgwSixpaalaaabaGaemyCae3aaSbaaSqaaiabdsfaunaaBaaameaacqWGRbWAcqGHRaWkcqaIXaqmaeqaaaWcbeaakiabcIcaOiab=HfaynaaCaaaleqabaGaeiikaGIaem4AaSMaeiykaKcaaOGaeiykaKcabaGaemyCae3aaSbaaSqaaiabdsfaunaaBaaameaacqWGRbWAcqGHRaWkcqaIXaqmaeqaaaWcbeaakiabcIcaOiab=HfaynaaCaaaleqabaGaeiikaGIaem4AaSMaey4kaSIaeGymaeJaeiykaKcaaOGaeiykaKcaaaGaay5Eaiaaw2haaaqaaiabg2da9iGbc2gaTjabcggaHjabcIha4naacmqabaGaeGymaeJaeiilaWIagiyzauMaeiiEaGNaeiiCaaNaei4waSLaeyOeI0IaeuiLdqecciGae4NSdi2aaSbaaSqaaiabdUgaRbqabaGccqqHuoarcqWGtbWucqGGDbqxaiaawUhacaGL9baacqGGSaalaaaaaa@92A4@

where, Δβk=1Tk+1−1Tk
 MathType@MTEF@5@5@+=feaafiart1ev1aaatCvAUfKttLearuWrP9MDH5MBPbIqV92AaeXatLxBI9gBaebbnrfifHhDYfgasaacH8akY=wiFfYdH8Gipec8Eeeu0xXdbba9frFj0=OqFfea0dXdd9vqai=hGuQ8kuc9pgc9s8qqaq=dirpe0xb9q8qiLsFr0=vr0=vr0dc8meaabaqaciaacaGaaeqabaqabeGadaaakeaacqqHuoariiGacqWFYoGydaWgaaWcbaGaem4AaSgabeaakiabg2da9maalaaabaGaeGymaedabaGaemivaq1aaSbaaSqaaiabdUgaRjabgUcaRiabigdaXaqabaaaaOGaeyOeI0YaaSaaaeaacqaIXaqmaeaacqWGubavdaWgaaWcbaGaem4AaSgabeaaaaaaaa@3C96@, Δ*S *= *S *(**X**^(*k *+ 1)^) - *S *(**X**^*k*^) and all weights are calculated with the configurations before the flip. This leads to a "random walk in temperature space" of the configurations.

Note that another possible approach based on Markov chains to compute p-values of a random model with a random variable *X*, ℙ [*X *> *b*] was introduced by Wilbur [45]. The first step is to sample from an unbiased Markov chain based on the model of interest and compute the median of the (high probability) distribution. In the second iteration the random walk is truncated such that only values larger than the median of the first iteration occur. This corresponds to choosing a lower temperaure *T *in Eq. (7). The third iteration uses the median of the second iteration and so forth. This is repeated until a fraction of 1/4 of all events lay beyond a certain threshold value leading to a non decreasing sequence of splitting intervals defined by the medians of each iteration. This sequence is used in the second stage of the algorithm, where p-values are computed explicitly by multiplying the p-values of the truncated distribution in each iteration.

Although this method is easy to implement and errors can be estimated relatively simply, the MCMCMC approach has the advantage that the different configurations are not subjected to a sequence of decreasing temperatures, but perform a random walk in temperature space, i.e. visit all temperatures several times. Thus, mixing is accelerated and hence fewer Monte Carlo steps are required.

### Reweighting the mixture

The production run of MCMCMC yield a set of *m *different chains of lengths *n*_*j*_. We denote the *ith *configuration in the chain of *jth *temperature as Xi(j)
 MathType@MTEF@5@5@+=feaafiart1ev1aaatCvAUfKttLearuWrP9MDH5MBPbIqV92AaeXatLxBI9gBaebbnrfifHhDYfgasaacH8akY=wiFfYdH8Gipec8Eeeu0xXdbba9frFj0=OqFfea0dXdd9vqai=hGuQ8kuc9pgc9s8qqaq=dirpe0xb9q8qiLsFr0=vr0=vr0dc8meaabaqaciaacaGaaeqabaqabeGadaaakeaaieqacqWFybawdaqhaaWcbaGaemyAaKgabaGaeiikaGIaemOAaOMaeiykaKcaaaaa@3282@. Of course this leads to a larger parameter range than simple importance reweighting of a single chain, hence Eq. (9) cannot be applied directly to the mixture. Geyer [46] developed a generalization of the importance reweighting formula to mixtures. His idea is based on Eq. (9), where *q*_*T *_is replaced by a "mixture weight" *q*_mix_, i.e. (using *q*_*j *_≡ q˜Tj
 MathType@MTEF@5@5@+=feaafiart1ev1aaatCvAUfKttLearuWrP9MDH5MBPbIqV92AaeXatLxBI9gBaebbnrfifHhDYfgasaacH8akY=wiFfYdH8Gipec8Eeeu0xXdbba9frFj0=OqFfea0dXdd9vqai=hGuQ8kuc9pgc9s8qqaq=dirpe0xb9q8qiLsFr0=vr0=vr0dc8meaabaqaciaacaGaaeqabaqabeGadaaakeaacuWGXbqCgaacamaaBaaaleaacqWGubavdaWgaaadbaGaemOAaOgabeaaaSqabaaaaa@3118@, i.e. *q*_*j *_represents the unormalized weights)

ET′[g(X)]≈1Z∑j=1m∑i=1njqT′(Xi(j))qmix(Xi(j))⋅g(Xi(j)).
 MathType@MTEF@5@5@+=feaafiart1ev1aaatCvAUfKttLearuWrP9MDH5MBPbIqV92AaeXatLxBI9gBaebbnrfifHhDYfgasaacH8akY=wiFfYdH8Gipec8Eeeu0xXdbba9frFj0=OqFfea0dXdd9vqai=hGuQ8kuc9pgc9s8qqaq=dirpe0xb9q8qiLsFr0=vr0=vr0dc8meaabaqaciaacaGaaeqabaqabeGadaaakeaatuuDJXwAK1uy0HMmaeHbfv3ySLgzG0uy0HgiuD3BaGabaiab=ri8fnaaBaaaleaacuWGubavgaqbaaqabaGccqGGBbWwcqWGNbWzcqGGOaakieqacqGFybawcqGGPaqkcqGGDbqxcqGHijYUdaWcaaqaaiabigdaXaqaaiabdQfaAbaadaaeWbqaamaaqahabaWaaSaaaeaacqWGXbqCdaWgaaWcbaGafmivaqLbauaaaeqaaOGaeiikaGIae4hwaG1aa0baaSqaaiabdMgaPbqaaiabcIcaOiabdQgaQjabcMcaPaaakiabcMcaPaqaaiabdghaXnaaBaaaleaacqqGTbqBcqqGPbqAcqqG4baEaeqaaOGaeiikaGIae4hwaG1aa0baaSqaaiabdMgaPbqaaiabcIcaOiabdQgaQjabcMcaPaaakiabcMcaPaaaaSqaaiabdMgaPjabg2da9iabigdaXaqaaiabd6gaUnaaBaaameaacqWGQbGAaeqaaaqdcqGHris5aaWcbaGaemOAaOMaeyypa0JaeGymaedabaGaemyBa0ganiabggHiLdGccqGHflY1cqWGNbWzcqGGOaakcqGFybawdaqhaaWcbaGaemyAaKgabaGaeiikaGIaemOAaOMaeiykaKcaaOGaeiykaKIaeiOla4caaa@7812@

The (global) normalization constant is given by Z=∑j=1m∑i=1njqT′(Xi(j))/qmix(Xi(j))
 MathType@MTEF@5@5@+=feaafiart1ev1aaatCvAUfKttLearuWrP9MDH5MBPbIqV92AaeXatLxBI9gBaebbnrfifHhDYfgasaacH8akY=wiFfYdH8Gipec8Eeeu0xXdbba9frFj0=OqFfea0dXdd9vqai=hGuQ8kuc9pgc9s8qqaq=dirpe0xb9q8qiLsFr0=vr0=vr0dc8meaabaqaciaacaGaaeqabaqabeGadaaakeaacqWGAbGwcqGH9aqpdaaeWaqaamaaqadabaGaemyCae3aaSbaaSqaaiqbdsfauzaafaaabeaaaeaacqWGPbqAcqGH9aqpcqaIXaqmaeaacqWGUbGBdaWgaaadbaGaemOAaOgabeaaa0GaeyyeIuoaaSqaaiabdQgaQjabg2da9iabigdaXaqaaiabd2gaTbqdcqGHris5aOGaeiikaGccbeGae8hwaG1aa0baaSqaaiabdMgaPbqaaiabcIcaOiabdQgaQjabcMcaPaaakiabcMcaPiabc+caViabdghaXnaaBaaaleaacqqGTbqBcqqGPbqAcqqG4baEaeqaaOGaeiikaGIae8hwaG1aa0baaSqaaiabdMgaPbqaaiabcIcaOiabdQgaQjabcMcaPaaakiabcMcaPaaa@568E@. The mixture weight function is known up to normalization constants cj≡ZTj
 MathType@MTEF@5@5@+=feaafiart1ev1aaatCvAUfKttLearuWrP9MDH5MBPbIqV92AaeXatLxBI9gBaebbnrfifHhDYfgasaacH8akY=wiFfYdH8Gipec8Eeeu0xXdbba9frFj0=OqFfea0dXdd9vqai=hGuQ8kuc9pgc9s8qqaq=dirpe0xb9q8qiLsFr0=vr0=vr0dc8meaabaqaciaacaGaaeqabaqabeGadaaakeaacqWGJbWydaWgaaWcbaGaemOAaOgabeaakiabggMi6kabdQfaAnaaBaaaleaacqWGubavdaWgaaadbaGaemOAaOgabeaaaSqabaaaaa@3586@:

qmix(X)=∑j=1mnjn⋅qj(X)cj,
 MathType@MTEF@5@5@+=feaafiart1ev1aaatCvAUfKttLearuWrP9MDH5MBPbIqV92AaeXatLxBI9gBaebbnrfifHhDYfgasaacH8akY=wiFfYdH8Gipec8Eeeu0xXdbba9frFj0=OqFfea0dXdd9vqai=hGuQ8kuc9pgc9s8qqaq=dirpe0xb9q8qiLsFr0=vr0=vr0dc8meaabaqaciaacaGaaeqabaqabeGadaaakeaacqWGXbqCdaWgaaWcbaGaeeyBa0MaeeyAaKMaeeiEaGhabeaakiabcIcaOGqabiab=HfayjabcMcaPiabg2da9maaqahabaWaaSaaaeaacqWGUbGBdaWgaaWcbaGaemOAaOgabeaaaOqaaiabd6gaUbaaaSqaaiabdQgaQjabg2da9iabigdaXaqaaiabd2gaTbqdcqGHris5aOGaeyyXIC9aaSaaaeaacqWGXbqCdaWgaaWcbaGaemOAaOgabeaakiabcIcaOiab=HfayjabcMcaPaqaaiabdogaJnaaBaaaleaacqWGQbGAaeqaaaaakiabcYcaSaaa@4DE5@

with *n *= ∑_*j*_*n*_*j*_. The unknown constants **c **≡ (*c*_1_, ..., *c*_*m*_) may be estimated by *reverse logistic regression *introduced by Geyer [46]. Here we used an alternative approach to obtain the constants **c **developed by Meng and Wong [47], which is explained now.

Since the global normalization constant *Z *in Eq. (11) is trivial, the problem is reduced to the estimation of (*m *- 1) ratios of normalization constants to some reference value. One possible choice is to fix the normalization constant of *q*_1 _and estimate the ratios *r*_*i *_= *c*_1_/*c*_*i *_(*i *= 2, ..., *m*).

**Figure 1:**
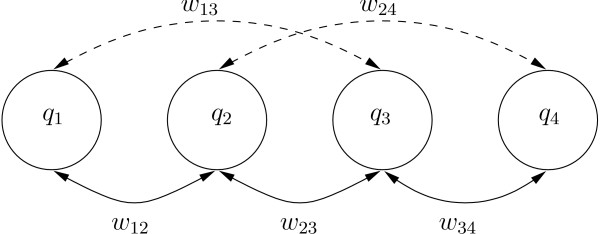
Sketch of the graph of overlapping distributions *q*_1_,..., *q*_4_. Distant distributions have weak overlaps.

Since the support of the mixture distribution is broader than each of the particular distributions, not all pairs of distributions *q*_*i *_and *q*_*j *_overlap in general. The overlaps of the empirical data can be measured by the matrix

wij=1ninj∑S(∑k=1nihS(Xk(i)))⋅(∑l=1njhS(Xl(j)))
 MathType@MTEF@5@5@+=feaafiart1ev1aaatCvAUfKttLearuWrP9MDH5MBPbIqV92AaeXatLxBI9gBaebbnrfifHhDYfgasaacH8akY=wiFfYdH8Gipec8Eeeu0xXdbba9frFj0=OqFfea0dXdd9vqai=hGuQ8kuc9pgc9s8qqaq=dirpe0xb9q8qiLsFr0=vr0=vr0dc8meaabaqaciaacaGaaeqabaqabeGadaaakeaacqWG3bWDdaWgaaWcbaGaemyAaKMaemOAaOgabeaakiabg2da9maalaaabaGaeGymaedabaGaemOBa42aaSbaaSqaaiabdMgaPbqabaGccqWGUbGBdaWgaaWcbaGaemOAaOgabeaaaaGcdaaeqbqaamaabmaabaWaaabCaeaacqWGObaAdaWgaaWcbaGaem4uamfabeaakiabcIcaOGqabiab=HfaynaaDaaaleaacqWGRbWAaeaacqGGOaakcqWGPbqAcqGGPaqkaaGccqGGPaqkaSqaaiabdUgaRjabg2da9iabigdaXaqaaiabd6gaUnaaBaaameaacqWGPbqAaeqaaaqdcqGHris5aaGccaGLOaGaayzkaaaaleaacqWGtbWuaeqaniabggHiLdGccqGHflY1daqadaqaamaaqahabaGaemiAaG2aaSbaaSqaaiabdofatbqabaGccqGGOaakcqWFybawdaqhaaWcbaGaemiBaWgabaGaeiikaGIaemOAaOMaeiykaKcaaOGaeiykaKcaleaacqWGSbaBcqGH9aqpcqaIXaqmaeaacqWGUbGBdaWgaaadbaGaemOAaOgabeaaa0GaeyyeIuoaaOGaayjkaiaawMcaaaaa@677B@

and the set of distributions can be represented by a graph (*V, E*) with vertices being the weight functions *V *= {*q*_1_, ..., *q*_*m*_} and the set of all overlaps being the weighted edges *E *= {*w*_*ij*_} with *w*_*ij *_> 0(see Fig. 1. We require, that the so constructed graph is connected. In practice one must find paths between each pair of distributions with not too small weights. In this case each distribution has a finite overlap with *q*_*mix *_and reweighting become possible on the full support.

Consider arbitrary weight functions *α*_*ij *_assigned to each edge of the graph and define the following expectation values with respect to *q*_*j*_

bji=Ej[qi(X)⋅αij(X)]=1cj∑Xqj(X)⋅qi(X)⋅αij(X)=cicjbij.
 MathType@MTEF@5@5@+=feaafiart1ev1aaatCvAUfKttLearuWrP9MDH5MBPbIqV92AaeXatLxBI9gBaebbnrfifHhDYfgasaacH8akY=wiFfYdH8Gipec8Eeeu0xXdbba9frFj0=OqFfea0dXdd9vqai=hGuQ8kuc9pgc9s8qqaq=dirpe0xb9q8qiLsFr0=vr0=vr0dc8meaabaqaciaacaGaaeqabaqabeGadaaakeaacqWGIbGydaWgaaWcbaGaemOAaOMaemyAaKgabeaakiabg2da9mrr1ngBPrwtHrhAYaqeguuDJXwAKbstHrhAGq1DVbaceaGae8hHWx0aaSbaaSqaaiabdQgaQbqabaGccqGGBbWwcqWGXbqCdaWgaaWcbaGaemyAaKgabeaakiabcIcaOGqabiab+HfayjabcMcaPiabgwSixJGaciab9f7aHnaaBaaaleaacqWGPbqAcqWGQbGAaeqaaOGaeiikaGIae4hwaGLaeiykaKIaeiyxa0Laeyypa0ZaaSaaaeaacqaIXaqmaeaacqWGJbWydaWgaaWcbaGaemOAaOgabeaaaaGcdaaeqbqaaiabdghaXnaaBaaaleaacqWGQbGAaeqaaOGaeiikaGIae4hwaGLaeiykaKIaeyyXICTaemyCae3aaSbaaSqaaiabdMgaPbqabaGccqGGOaakcqGFybawcqGGPaqkcqGHflY1cqqFXoqydaWgaaWcbaGaemyAaKMaemOAaOgabeaakiabcIcaOiab+HfayjabcMcaPaWcbaGae4hwaGfabeqdcqGHris5aOGaeyypa0ZaaSaaaeaacqWGJbWydaWgaaWcbaGaemyAaKgabeaaaOqaaiabdogaJnaaBaaaleaacqWGQbGAaeqaaaaakiabdkgaInaaBaaaleaacqWGPbqAcqWGQbGAaeqaaOGaeiOla4caaa@7D94@

This means, for any given vector **c**, all values {*b*_*ji*_} can be calculated using this expression. We require the *α*_*ij *_to be symmetric, i.e. *α*_*ij *_= *α*_*ji*_, and a finite overlap with each of the distributions. With *r*_1 _= 1 and *r*_*i*_*b*_*ji *_= *r*_*j*_*b*_*ij *_it is straight forward to construct a linear system for the remaining (*m *- 1) ratios, for *i *> 1:

bi1=b1i⋅ri=∑j≠ibji⋅ri−∑j≠i,j>1bij⋅rj≡∑j>1aij⋅rj,
 MathType@MTEF@5@5@+=feaafiart1ev1aaatCvAUfKttLearuWrP9MDH5MBPbIqV92AaeXatLxBI9gBaebbnrfifHhDYfgasaacH8akY=wiFfYdH8Gipec8Eeeu0xXdbba9frFj0=OqFfea0dXdd9vqai=hGuQ8kuc9pgc9s8qqaq=dirpe0xb9q8qiLsFr0=vr0=vr0dc8meaabaqaciaacaGaaeqabaqabeGadaaakeaacqWGIbGydaWgaaWcbaGaemyAaKMaeGymaedabeaakiabg2da9iabdkgaInaaBaaaleaacqaIXaqmcqWGPbqAaeqaaOGaeyyXICTaemOCai3aaSbaaSqaaiabdMgaPbqabaGccqGH9aqpdaaeqbqaaiabdkgaInaaBaaaleaacqWGQbGAcqWGPbqAaeqaaaqaaiabdQgaQjabgcMi5kabdMgaPbqab0GaeyyeIuoakiabgwSixlabdkhaYnaaBaaaleaacqWGPbqAaeqaaOGaeyOeI0YaaabuaeaacqWGIbGydaWgaaWcbaGaemyAaKMaemOAaOgabeaakiabgwSixlabdkhaYnaaBaaaleaacqWGQbGAaeqaaaqaaiabdQgaQjabgcMi5kabdMgaPjabcYcaSiabdQgaQjabg6da+iabigdaXaqab0GaeyyeIuoakiabggMi6oaaqafabaGaemyyae2aaSbaaSqaaiabdMgaPjabdQgaQbqabaGccqGHflY1cqWGYbGCdaWgaaWcbaGaemOAaOgabeaaaeaacqWGQbGAcqGH+aGpcqaIXaqmaeqaniabggHiLdGccqGGSaalaaa@728D@

with *a*_*ii *_= ∑_*j *≠ *i *_*b*_*ij *_and *a*_*ij *_= -*b*_*ij *_for *i *≠ *j*. This equations cannot be solved directly, because the coefficients *a*_*ij *_do depend on the unknown ratios. However it is possible to solve Eq. (13) self-consistently. Using b^
 MathType@MTEF@5@5@+=feaafiart1ev1aaatCvAUfKttLearuWrP9MDH5MBPbIqV92AaeXatLxBI9gBaebbnrfifHhDYfgasaacH8akY=wiFfYdH8Gipec8Eeeu0xXdbba9frFj0=OqFfea0dXdd9vqai=hGuQ8kuc9pgc9s8qqaq=dirpe0xb9q8qiLsFr0=vr0=vr0dc8meaabaqaciaacaGaaeqabaqabeGadaaakeaaieqacuWFIbGygaqcaaaa@2E0F@ = (*b*_11_, *b*_21_,..., *b*_*m*1_) and including explicitely the dependence on **r **= (*r*_1_, *r*_2_,..., *r*_*m*_) we obtain

*A *(**r**^(*t*)^)·**r**^(*t *+ 1) ^= **b**(**r**^(*t*)^).

This equation can be solved by starting with **r**^(1) ^= (1, 1, ..., 1) and iteratively solving for **r**^(*t *+ 1) ^till convergence. Following the paper of Meng and Wong [47] Eq. (14) with the choice αij(X)=ninj|n|2⋅qmix(X)
 MathType@MTEF@5@5@+=feaafiart1ev1aaatCvAUfKttLearuWrP9MDH5MBPbIqV92AaeXatLxBI9gBaebbnrfifHhDYfgasaacH8akY=wiFfYdH8Gipec8Eeeu0xXdbba9frFj0=OqFfea0dXdd9vqai=hGuQ8kuc9pgc9s8qqaq=dirpe0xb9q8qiLsFr0=vr0=vr0dc8meaabaqaciaacaGaaeqabaqabeGadaaakeaaiiGacqWFXoqydaWgaaWcbaGaemyAaKMaemOAaOgabeaakiabcIcaOGqabiab+HfayjabcMcaPiabg2da9maalaaabaGaemOBa42aaSbaaSqaaiabdMgaPbqabaGccqWGUbGBdaWgaaWcbaGaemOAaOgabeaaaOqaamaaemaabaGaemOBa4gacaGLhWUaayjcSdWaaWbaaSqabeaacqaIYaGmaaaaaOGaeyyXICTaemyCae3aaSbaaSqaaiabb2gaTjabbMgaPjabbIha4bqabaGccqGGOaakcqGFybawcqGGPaqkaaa@4BE6@ converges to same estimator as proposed by Geyer [46], which is based on maximization of a quasi-loglikelihood. The desired probability *P *(*s*) can be achieved by setting *q*_*T' *_to the unbiased weight *q*_∞ _= 1 and estimate the expectation values of the indicator functions *h*_*S *_in Eq. (11).

### Illustration and convergence diagnostics

In order to guarantee start configurations taken from the stationary distribution the first few iterations of the chains have to be discarded. The number of iterations to be discarded is denoted as burning or equilibration period. Usually one starts from a random (i.e. disordered) configuration and equilibrates the system. At the beginning of the simulation the system has a low score and hence it can reach in principle most regions of the score landscape. If the temperature is low, one sees when looking at Eq. (7) that configurations with large score dominate. Hence, typically the score increases or stays the same during the simulation with only few score-decreasing fluctuations.

Note that if "ground states" are also known, i.e. the maxima of the score landscape, the reverse process is possible, i.e. starting from a high maximum and sampling its local environment. One can use this fact to verify, whether a system has equilibrated on a larger scale, i.e. whether it is able to overcome the typical barriers in the score landscape. This is the case when the average behavior for two runs, one starting with a disordered configuration and one starting with an "ground-state" configuration, is the same (within fluctuation). If the temperature is too small, this is usually not possible.

It is helpful to consider a simple toy system to illustrate and benchmark the method, in detail consider a 4-letter alphabet of equal weights and sequence lengths *L *= *M *= 10, 20. The scoring system is defined by the score matrix

σ(a,b)={+1if a=b−3else.
 MathType@MTEF@5@5@+=feaafiart1ev1aaatCvAUfKttLearuWrP9MDH5MBPbIqV92AaeXatLxBI9gBaebbnrfifHhDYfgasaacH8akY=wiFfYdH8Gipec8Eeeu0xXdbba9frFj0=OqFfea0dXdd9vqai=hGuQ8kuc9pgc9s8qqaq=dirpe0xb9q8qiLsFr0=vr0=vr0dc8meaabaqaciaacaGaaeqabaqabeGadaaakeaaiiGacqWFdpWCcqGGOaakcqWGHbqycqGGSaalcqWGIbGycqGGPaqkcqGH9aqpdaGabeqaauaabaqaciaaaeaacqGHRaWkcqaIXaqmaeaacqqGPbqAcqqGMbGzcqqGGaaicqWGHbqycqGH9aqpcqWGIbGyaeaacqGHsislcqaIZaWmaeaacqWGLbqzcqWGSbaBcqWGZbWCcqWGLbqzaaaacaGL7baacqGGUaGlaaa@46EE@

and affine gap costs with *α *= 4 and *β *= 2.

**Figure 2:**
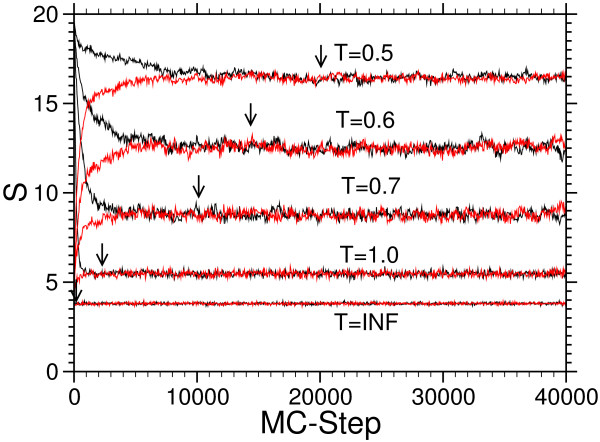
Equilibration of the 4-letter system (*L *= *M *= 20) with temperatures *T *= 0.5, 0.6, 0.7, 1.0, ∞ Equilibrium is reached after 20000, 15000, 10000, 1000, 100 steps (indicated by arrows) respectively. *S *(*t*) is averaged over independent 250 runs.

An illustration of the equilibration criterion is given in Fig. 2. By "visual inspection" we obtain equilibration times 100 (*T *= ∞),1000 (*T *= 1), 10000 (*T *= 0.7), 15000 (*T *= 0.6) and 20000 (*T *= 0.5), respectively.

A more quantitative method was introduced by Raftery and Lewis [48,49], that estimates equilibration and sample times for a set of quantils. Raftery and Lewis's program, which is available from *StatLib *[50] or in the *CODA *package [51], estimates a *thining interval n*_thin _as well. That means only every *n*_thin_*th *step is used for inference in order to avoid correlations between the scores at time *t *and *t *+ Δ*t*, that occur in MCMC in constrast to direct generating random sequences. The program requires three parameters: the desired accuracy *r*, the required probability *s *of attaining the specified accuracy and a less relevant tolerance parameter *ε*.

We compared the result of the estimate of the equilibration time with the simple visual approach: For the example given in Fig. 2 we maximized numerical estimate of equilibration time over a set of quantils between 0.1 and 0.95 for *r *= 0.0125, *s *= 0.95, *ε *= 0.001): The results for the equilibration time obtained by this approach are always much smaller than those obtained by the visual inspection. For example for *L *= 20, the Rafter-Lewis approach gives an equilibration time of 800 steps for the lowest temperature, whereas Fig. 2 suggests 20000 steps. Therefore equilibrium might not be guaranteed with the Rafter-Lewis approach and the visual inspection seems to be more conservative.

To estimate the times scales over which the simulation decorrelates, we considered the autocorrelation function

ξ(t)=〈S(t0)S(t0+t)〉t0−〈S(t0)〉t02〈S(t0)2〉t0−〈S(t0)〉t02,
 MathType@MTEF@5@5@+=feaafiart1ev1aaatCvAUfKttLearuWrP9MDH5MBPbIqV92AaeXatLxBI9gBaebbnrfifHhDYfgasaacH8akY=wiFfYdH8Gipec8Eeeu0xXdbba9frFj0=OqFfea0dXdd9vqai=hGuQ8kuc9pgc9s8qqaq=dirpe0xb9q8qiLsFr0=vr0=vr0dc8meaabaqaciaacaGaaeqabaqabeGadaaakeaaiiGacqWF+oaEcqGGOaakcqWG0baDcqGGPaqkcqGH9aqpdaWcaaqaamaaamqabaGaem4uamLaeiikaGIaemiDaq3aaSbaaSqaaiabicdaWaqabaGccqGGPaqkcqWGtbWucqGGOaakcqWG0baDdaWgaaWcbaGaeGimaadabeaakiabgUcaRiabdsha0jabcMcaPaGaayzkJiaawQYiamaaBaaaleaacqWG0baDdaWgaaadbaGaeGimaadabeaaaSqabaGccqGHsisldaaadeqaaiabdofatjabcIcaOiabdsha0naaBaaaleaacqaIWaamaeqaaOGaeiykaKcacaGLPmIaayPkJaWaa0baaSqaaiabdsha0naaBaaameaacqaIWaamaeqaaaWcbaGaeGOmaidaaaGcbaWaaaWabeaacqWGtbWucqGGOaakcqWG0baDdaWgaaWcbaGaeGimaadabeaakiabcMcaPmaaCaaaleqabaGaeGOmaidaaaGccaGLPmIaayPkJaWaaSbaaSqaaiabdsha0naaBaaameaacqaIWaamaeqaaaWcbeaakiabgkHiTmaaamqabaGaem4uamLaeiikaGIaemiDaq3aaSbaaSqaaiabicdaWaqabaGccqGGPaqkaiaawMYicaGLQmcadaqhaaWcbaGaemiDaq3aaSbaaWqaaiabicdaWaqabaaaleaacqaIYaGmaaaaaOGaeiilaWcaaa@6891@

**Figure 3:**
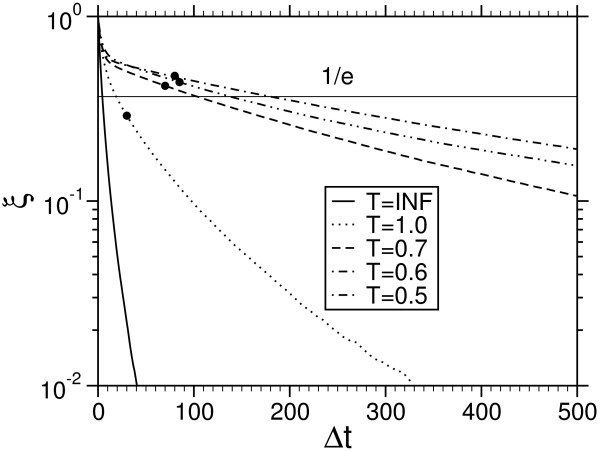
Score auto-correlation function for different temperatures (4 letters, *L *= *M *= 20). Circles indicate corre-sponding *n*_thin _from Raftery and Lewis [48,49].

〈⋯〉t0
 MathType@MTEF@5@5@+=feaafiart1ev1aaatCvAUfKttLearuWrP9MDH5MBPbIqV92AaeXatLxBI9gBaebbnrfifHhDYfgasaacH8akY=wiFfYdH8Gipec8Eeeu0xXdbba9frFj0=OqFfea0dXdd9vqai=hGuQ8kuc9pgc9s8qqaq=dirpe0xb9q8qiLsFr0=vr0=vr0dc8meaabaqaciaacaGaaeqabaqabeGadaaakeaadaaadeqaaiabl+UimbGaayzkJiaawQYiamaaBaaaleaacqWG0baDdaWgaaadbaGaeGimaadabeaaaSqabaaaaa@332E@ denoting the average over different times and independent runs. The typical time scale, over which correlation vanish is the correlation time *τ *defined via *ξ *(*τ*)= 1/*e*. The normalized auto-correlation function for the system of *L *= 20 is shown in Fig. 3. A comparison with Raftery and Lewis diagnostics of *n*_thin_, indicated by dots, gives evidence that the two estimates coincide with each other at least in the order of magnitude. The correlation time increases with decreasing temperature, which corresponds to a growth of the equilibration time with decreasing temperature in Fig. 2. However by the generation of the histograms the correlations will average out, but estimates of the errors are more complicated when the data are correlated. However the consideration of *τ *and *n*_thin _has some practical issues too: For the application it is only necessary to infere every 100 *th *step, which saves a lot disk space.

Once the equilibration period is estimated one may check the convergence of the remaining parts of the chains to the equilibrium distributions. This was done by computing the Gelman and Rubin shrink factors *R *[49,52,53]. This diagnostic compares the "within-chain" and the "inter-chain variance" of a set of multiple Monte Carlo chains. When the factor *R *approaches 1 the within-chain variance dominates and the sampler has forgotten its starting point. For the lowest temperature in our toy model *L *= 20 we found *R *= 1.03 for the 99.995% quantile, which appears to be reasonable.

**Figure 4:**
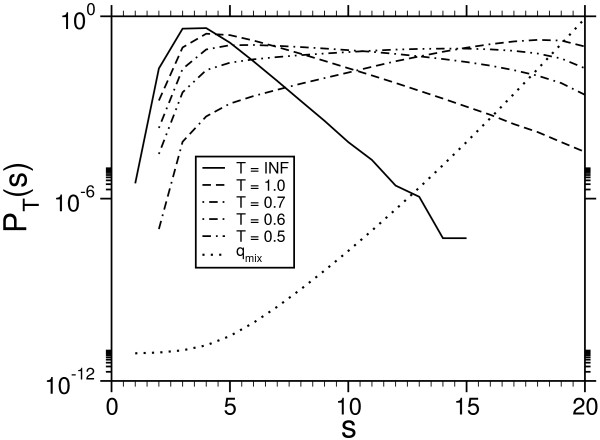
Empirical probabilities for the toy model (4 letters, *L *= *M *= 20) held at finite temperature. The dottet line showes the normalized mixture weight function q^mix
 MathType@MTEF@5@5@+=feaafiart1ev1aaatCvAUfKttLearuWrP9MDH5MBPbIqV92AaeXatLxBI9gBaebbnrfifHhDYfgasaacH8akY=wiFfYdH8Gipec8Eeeu0xXdbba9frFj0=OqFfea0dXdd9vqai=hGuQ8kuc9pgc9s8qqaq=dirpe0xb9q8qiLsFr0=vr0=vr0dc8meaabaqaciaacaGaaeqabaqabeGadaaakeaacuWGXbqCgaqcamaaBaaaleaacqqGTbqBcqqGPbqAcqqG4baEaeqaaaaa@3284@.

From the equilibrated and converged chains we obtained histograms for different temperatures, which are shown in Fig. 4 for the case *L *= 20.

The empirical overlap matrix of this mixture is estimated by

(wij)≈(10.5430.2560.0980.0090.54310.5720.2660.0700.2560.57210.6240.2640.0980.2660.62410.5700.0090.0700.2640.5701),
 MathType@MTEF@5@5@+=feaafiart1ev1aaatCvAUfKttLearuWrP9MDH5MBPbIqV92AaeXatLxBI9gBaebbnrfifHhDYfgasaacH8akY=wiFfYdH8Gipec8Eeeu0xXdbba9frFj0=OqFfea0dXdd9vqai=hGuQ8kuc9pgc9s8qqaq=dirpe0xb9q8qiLsFr0=vr0=vr0dc8meaabaqaciaacaGaaeqabaqabeGadaaakeaacqGGOaakcqWG3bWDdaWgaaWcbaGaemyAaKMaemOAaOgabeaakiabcMcaPiabgIKi7oaabmaabaqbaeqabuqbaaaaaeaacqaIXaqmaeaacqaIWaamcqGGUaGlcqaI1aqncqaI0aancqaIZaWmaeaacqaIWaamcqGGUaGlcqaIYaGmcqaI1aqncqaI2aGnaeaacqaIWaamcqGGUaGlcqaIWaamcqaI5aqocqaI4aaoaeaacqaIWaamcqGGUaGlcqaIWaamcqaIWaamcqaI5aqoaeaacqaIWaamcqGGUaGlcqaI1aqncqaI0aancqaIZaWmaeaacqaIXaqmaeaacqaIWaamcqGGUaGlcqaI1aqncqaI3aWncqaIYaGmaeaacqaIWaamcqGGUaGlcqaIYaGmcqaI2aGncqaI2aGnaeaacqaIWaamcqGGUaGlcqaIWaamcqaI3aWncqaIWaamaeaacqaIWaamcqGGUaGlcqaIYaGmcqaI1aqncqaI2aGnaeaacqaIWaamcqGGUaGlcqaI1aqncqaI3aWncqaIYaGmaeaacqaIXaqmaeaacqaIWaamcqGGUaGlcqaI2aGncqaIYaGmcqaI0aanaeaacqaIWaamcqGGUaGlcqaIYaGmcqaI2aGncqaI0aanaeaacqaIWaamcqGGUaGlcqaIWaamcqaI5aqocqaI4aaoaeaacqaIWaamcqGGUaGlcqaIYaGmcqaI2aGncqaI2aGnaeaacqaIWaamcqGGUaGlcqaI2aGncqaIYaGmcqaI0aanaeaacqaIXaqmaeaacqaIWaamcqGGUaGlcqaI1aqncqaI3aWncqaIWaamaeaacqaIWaamcqGGUaGlcqaIWaamcqaIWaamcqaI5aqoaeaacqaIWaamcqGGUaGlcqaIWaamcqaI3aWncqaIWaamaeaacqaIWaamcqGGUaGlcqaIYaGmcqaI2aGncqaI0aanaeaacqaIWaamcqGGUaGlcqaI1aqncqaI3aWncqaIWaamaeaacqaIXaqmaaaacaGLOaGaayzkaaGaeiilaWcaaa@99D0@

which has a finite overlap between *all *pairs. Note that in general a weaker condition must be fulfilled, namely that a connected path from the lowest to the hightest temperature must be possible, as outlined before. In more complex models only this condidition might be fulfilled.

**Figure 5:**
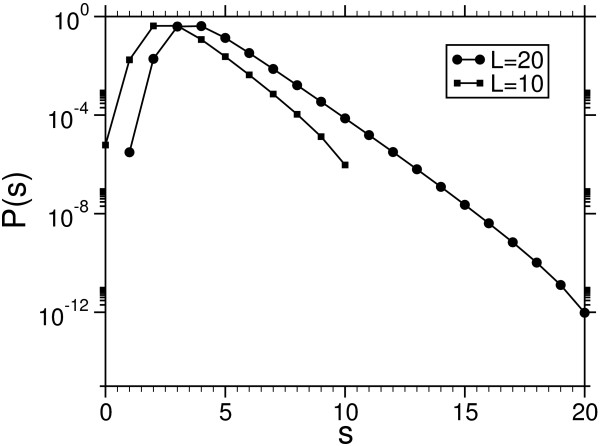
Score probabilities obtained throw the reweighting mixture technique for a 4-letter system with sequence-length *L *= 10, 20 and scoring parameters Eq. (15) using affine gap costs (*α *= 4, *β *= 2). For *L *= 10 the *P *(*s*) had also been been obtained by exact enumeration of all 4^2 × 10 ^configurations. A difference between the empirical curve is not visible in the plot.

Applying the reweighting technique, which was explained in the previous section, we obtain the infinite temperature probability *P *(*s*) (see Fig. 5).

**Figure 6:**
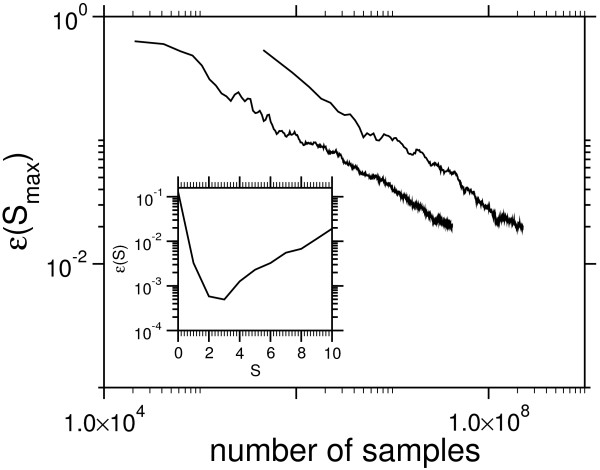
Rate of convergence of the MCMCMC data. The relative error *ε *(*S*_max_) of the ground state for *L *= 10 and *L *= 20 depending on the number *N*_samples _of samples is shown. Inset: relative error of the final *P *(*s*) incomparison to the exact enumeration of all states for the smallest system *L *= 10.

Obviously, the toy model has *Z *= 4^2 *L *^configurations. The maximum score over the ensemble of all possible configurations is *S*_max _= *L*. This corresponds to a pair of sequences with *L *equal letters *x*_*i *_= *y*_*i *_(*i *= 1 ... *L*). The number of configurations with the highest score is 4^*L*^. Hence, the probability to find a maximum score among all random sequences is *P *(*S*_max_) = ℙ[*S *= *S*_max_] = 4^*L*^/4^2 *L *^= 4^-*L*^. Below, to benchmark the Monte Carlo algorithm, we compare the convergence of the relative error ε(Smax⁡)=|Psample(Smax⁡)−4−L|4−L
 MathType@MTEF@5@5@+=feaafiart1ev1aaatCvAUfKttLearuWrP9MDH5MBPbIqV92AaeXatLxBI9gBaebbnrfifHhDYfgasaacH8akY=wiFfYdH8Gipec8Eeeu0xXdbba9frFj0=OqFfea0dXdd9vqai=hGuQ8kuc9pgc9s8qqaq=dirpe0xb9q8qiLsFr0=vr0=vr0dc8meaabaqaciaacaGaaeqabaqabeGadaaakeaaiiGacqWF1oqzcqGGOaakcqWGtbWudaWgaaWcbaGagiyBa0MaeiyyaeMaeiiEaGhabeaakiabcMcaPiabg2da9maalaaabaWaaqWaaeaacqWGqbaudaWgaaWcbaGaee4CamNaeeyyaeMaeeyBa0MaeeiCaaNaeeiBaWMaeeyzaugabeaakiabcIcaOiabdofatnaaBaaaleaacyGGTbqBcqGGHbqycqGG4baEaeqaaOGaeiykaKIaeyOeI0IaeGinaqZaaWbaaSqabeaacqGHsislcqWGmbataaaakiaawEa7caGLiWoaaeaacqaI0aandaahaaWcbeqaaiabgkHiTiabdYeambaaaaaaaa@51F2@ for different sequence lengths, *P*_sample _(*s*) being the corresponding probability obtained from the MC simulation. From Fig. 6, which illustrates convergence of the *ε *(*S*_max_) as a function of total sample size for all temperatures. In order to get a clear picture we averaged over several blocks of runs.

For small systems one may enumerate all possible configurations and compare the complete distribution with the Monte Carlo data. The empirical probability distribution for *L *= 10 in Fig. 5 coincides with the exact result, such that a the difference is not visible in the plot. However *L *= 10 is a very small system in contrast to real biological sequences, which are considered in section "Results", but exact enumeration is only possible on a modern computer cluster. Hence only for *L *= 10 the relative error ε(s)=|Psample(s)−Pexact(s)|Pexact(s)
 MathType@MTEF@5@5@+=feaafiart1ev1aaatCvAUfKttLearuWrP9MDH5MBPbIqV92AaeXatLxBI9gBaebbnrfifHhDYfgasaacH8akY=wiFfYdH8Gipec8Eeeu0xXdbba9frFj0=OqFfea0dXdd9vqai=hGuQ8kuc9pgc9s8qqaq=dirpe0xb9q8qiLsFr0=vr0=vr0dc8meaabaqaciaacaGaaeqabaqabeGadaaakeaaiiGacqWF1oqzcqGGOaakcqWGZbWCcqGGPaqkcqGH9aqpdaWcaaqaamaaemaabaGaemiuaa1aaSbaaSqaaiabbohaZjabbggaHjabb2gaTjabbchaWjabbYgaSjabbwgaLbqabaGccqGGOaakcqWGZbWCcqGGPaqkcqGHsislcqWGqbaudaWgaaWcbaGaeeyzauMaeeiEaGNaeeyyaeMaee4yamMaeeiDaqhabeaakiabcIcaOiabdohaZjabcMcaPaGaay5bSlaawIa7aaqaaiabdcfaqnaaBaaaleaacqqGLbqzcqqG4baEcqqGHbqycqqGJbWycqqG0baDaeqaaOGaeiikaGIaem4CamNaeiykaKcaaaaa@59E8@ (see inset of Fig. 6) can be computed on the full support. In principle one is able to reduce variance on the low score end of the distribution by introducing negative temperature values, but this is beyond of the scope of this article.

### Error estimation

As mentioned previously, a direct calculation of the errors is hardly possible. The first reason is that the Markov chain data are correlated. Secondly, the iterative estimation of the relative normalization constants is not trivial and contributes also to the overall error. Nevertheless, one can evaluate errors using the jackknife method [54]: First, in order to ensure, that the data are uncorrelated, we took data points which are seperated by at least the correlation time, determined via Eq. (16). Next, the dataset is divided into *n*_*b *_blocks of equal size (hence, the number should be a multiple of *n*_*b*_). Quantities of interests *g *are calculated *k *times (*k *= 1 ... *n*_*b*_), each time omitting block *B*_*k*_. These *n*_*b *_values are averaged over all possibilities of *k*, in the notation of Eq. (11)

〈g(X1,...,Xn)〉kJ=1Znb∑k=1nb∑j=1m∑i=1,i∉BknjqT′(Xi(j))qmix(Xi(j))⋅g(Xi(j)).
 MathType@MTEF@5@5@+=feaafiart1ev1aaatCvAUfKttLearuWrP9MDH5MBPbIqV92AaeXatLxBI9gBaebbnrfifHhDYfgasaacH8akY=wiFfYdH8Gipec8Eeeu0xXdbba9frFj0=OqFfea0dXdd9vqai=hGuQ8kuc9pgc9s8qqaq=dirpe0xb9q8qiLsFr0=vr0=vr0dc8meaabaqaciaacaGaaeqabaqabeGadaaakeaadaaadeqaaiabdEgaNjabcIcaOGqabiab=HfaynaaBaaaleaacqaIXaqmaeqaaOGaeiilaWIaeiOla4IaeiOla4IaeiOla4IaeiilaWIae8hwaG1aaSbaaSqaaiabd6gaUbqabaGccqGGPaqkaiaawMYicaGLQmcadaqhaaWcbaGaem4AaSgabaGaemOsaOeaaOGaeyypa0ZaaSaaaeaacqaIXaqmaeaacqWGAbGwcqWGUbGBdaWgaaWcbaGaemOyaigabeaaaaGcdaaeWbqaamaaqahabaWaaabCaeaadaWcaaqaaiabdghaXnaaBaaaleaacuWGubavgaqbaaqabaGccqGGOaakcqWFybawdaqhaaWcbaGaemyAaKgabaGaeiikaGIaemOAaOMaeiykaKcaaOGaeiykaKcabaGaemyCae3aaSbaaSqaaiabb2gaTjabbMgaPjabbIha4bqabaGccqGGOaakcqWFybawdaqhaaWcbaGaemyAaKgabaGaeiikaGIaemOAaOMaeiykaKcaaOGaeiykaKcaaaWcbaGaemyAaKMaeyypa0JaeGymaeJaeiilaWIaemyAaKMaeyycI8SaemOqai0aaSbaaWqaaiabdUgaRbqabaaaleaacqWGUbGBdaWgaaadbaGaemOAaOgabeaaa0GaeyyeIuoaaSqaaiabdQgaQjabg2da9iabigdaXaqaaiabd2gaTbqdcqGHris5aaWcbaGaem4AaSMaeyypa0JaeGymaedabaGaemOBa42aaSbaaWqaaiabdkgaIbqabaaaniabggHiLdGccqGHflY1cqWGNbWzcqGGOaakcqWFybawdaqhaaWcbaGaemyAaKgabaGaeiikaGIaemOAaOMaeiykaKcaaOGaeiykaKIaeiOla4caaa@8641@

The error of *g *is estimated by

σgJ=(nb−1)(〈g2(X1,...,Xn)〉kJ−(〈g(X1,...,Xn)〉kJ)2).
 MathType@MTEF@5@5@+=feaafiart1ev1aaatCvAUfKttLearuWrP9MDH5MBPbIqV92AaeXatLxBI9gBaebbnrfifHhDYfgasaacH8akY=wiFfYdH8Gipec8Eeeu0xXdbba9frFj0=OqFfea0dXdd9vqai=hGuQ8kuc9pgc9s8qqaq=dirpe0xb9q8qiLsFr0=vr0=vr0dc8meaabaqaciaacaGaaeqabaqabeGadaaakeaaiiGacqWFdpWCdaWgaaWcbaGaem4zaC2aaWbaaWqabeaacqWGkbGsaaaaleqaaOGaeyypa0ZaaOaaaeaacqGGOaakcqWGUbGBdaWgaaWcbaGaemOyaigabeaakiabgkHiTiabigdaXiabcMcaPmaabmaabaWaaaWabeaacqWGNbWzdaahaaWcbeqaaiabikdaYaaakiabcIcaOGqabiab+HfaynaaBaaaleaacqaIXaqmaeqaaOGaeiilaWIaeiOla4IaeiOla4IaeiOla4IaeiilaWIae4hwaG1aaSbaaSqaaiabd6gaUbqabaGccqGGPaqkaiaawMYicaGLQmcadaqhaaWcbaGaem4AaSgabaGaemOsaOeaaOGaeyOeI0YaaeWaaeaadaaadeqaaiabdEgaNjabcIcaOiab+HfaynaaBaaaleaacqaIXaqmaeqaaOGaeiilaWIaeiOla4IaeiOla4IaeiOla4IaeiilaWIae4hwaG1aaSbaaSqaaiabd6gaUbqabaGccqGGPaqkaiaawMYicaGLQmcadaqhaaWcbaGaem4AaSgabaGaemOsaOeaaaGccaGLOaGaayzkaaWaaWbaaSqabeaacqaIYaGmaaaakiaawIcacaGLPaaaaSqabaGccqGGUaGlaaa@627E@

For example the relative errors σrjJ/rj
 MathType@MTEF@5@5@+=feaafiart1ev1aaatCvAUfKttLearuWrP9MDH5MBPbIqV92AaeXatLxBI9gBaebbnrfifHhDYfgasaacH8akY=wiFfYdH8Gipec8Eeeu0xXdbba9frFj0=OqFfea0dXdd9vqai=hGuQ8kuc9pgc9s8qqaq=dirpe0xb9q8qiLsFr0=vr0=vr0dc8meaabaqaciaacaGaaeqabaqabeGadaaakeaaiiGacqWFdpWCdaWgaaWcbaGaemOCai3aa0baaWqaaiabdQgaQbqaaiabdQeakbaaaSqabaGccqGGVaWlcqWGYbGCdaWgaaWcbaGaemOAaOgabeaaaaa@36A8@ of the normalization constant ratios increase from 8.6 × 10^-4 ^for *r*_2 _to 1.29 × 10^-2 ^for *r*_5_. This indicates that the method is able to capture the error propagation of the relative normalization constants due to weak overlaps of distant distributions (see also Eq. (17)). Similar errors for the probabilities *P *(*s*) can be estimated by applying this approach.

## Results

### Optimal alignment statistics

Next, we show the results from the application of the method to biologically relevant systems: local sequence alignment of protein sequences using BLOSUM62 [20] and PAM250 [21,22] matrices. We apply amino acid background frequencies by Robinson and Robinson [55]. We consider different affine gap cost with 10 ≤ *α *≤ 16, *β *= 1 for the BLOSUM62 matrix and 11 ≤ *α *≤ 17, *β *= 3 when using the PAM250 matrix, as well as infinite gap costs. We study ten different sequence lengths between *M *= *L *= 40 and *M *= *L *= 400, in detail *L *= 40, 60, 80, 100, 150, 200, 250, 300, 350, 400.

Since the complexity of this system is much larger than the simple 4-letter system, the ground states could not be reached. Only temperatures where equilibration was guaranteed within a reasonable computation time were used for the calculation of *P *(*s*). This means that we cannot resolve the score probability distribution over its full support. But the range of temperatures is large enough to evaluate the distributions down to values *P *(*s*) ~10^-60^. The temperature sets we have used in the MCMCMC technique were varied between {2.00, 2.25, 2.50, 3.00, 5.00, 7.00, ∞} (*L *= 40) and {3.25, 3.50, 4.00, 5.00, 7.00, ∞} (*L *= 400) for BLOSUM62 matrices and between {2.75, 3.00, 3.25, 4.00, 5.00, 7.00, ∞} and {4.00, 4.25, 4.50, 5.00, 8.00, ∞} for the PAM250 matrices. For each run we performed 8 × 10^5 ^Monte Carlo steps. The Gelman and Rubin shrink factors fell below 1.04 in almost all cases. For BLOSUM62 matrices and *L *= 350, 400 a slightly longer run (10^6^) had been required to reduce *R*. The resulting probabilities were obtained from averaging over 10 (*L *= 400) up to 100 (*L *= 40) runs. The typical overlap matrix for the most complex system (*L *= 400, BLOSUM62) was

(wij)=(10.68500.50170.27170.04800.00150.685010.78570.46240.09840.00340.50170.785710.64090.16070.01170.27170.46240.640910.35870.05490.04800.09840.16070.358710.37770.00150.00340.01170.37770.37771).
 MathType@MTEF@5@5@+=feaafiart1ev1aaatCvAUfKttLearuWrP9MDH5MBPbIqV92AaeXatLxBI9gBaebbnrfifHhDYfgasaacH8akY=wiFfYdH8Gipec8Eeeu0xXdbba9frFj0=OqFfea0dXdd9vqai=hGuQ8kuc9pgc9s8qqaq=dirpe0xb9q8qiLsFr0=vr0=vr0dc8meaabaqaciaacaGaaeqabaqabeGadaaakeaacqGGOaakcqWG3bWDdaWgaaWcbaGaemyAaKMaemOAaOgabeaakiabcMcaPiabg2da9maabmaabaqbaeqabyGbaaaaaeaacqaIXaqmaeaacqaIWaamcqGGUaGlcqaI2aGncqaI4aaocqaI1aqncqaIWaamaeaacqaIWaamcqGGUaGlcqaI1aqncqaIWaamcqaIXaqmcqaI3aWnaeaacqaIWaamcqGGUaGlcqaIYaGmcqaI3aWncqaIXaqmcqaI3aWnaeaacqaIWaamcqGGUaGlcqaIWaamcqaI0aancqaI4aaocqaIWaamaeaacqaIWaamcqGGUaGlcqaIWaamcqaIWaamcqaIXaqmcqaI1aqnaeaacqaIWaamcqGGUaGlcqaI2aGncqaI4aaocqaI1aqncqaIWaamaeaacqaIXaqmaeaacqaIWaamcqGGUaGlcqaI3aWncqaI4aaocqaI1aqncqaI3aWnaeaacqaIWaamcqGGUaGlcqaI0aancqaI2aGncqaIYaGmcqaI0aanaeaacqaIWaamcqGGUaGlcqaIWaamcqaI5aqocqaI4aaocqaI0aanaeaacqaIWaamcqGGUaGlcqaIWaamcqaIWaamcqaIZaWmcqaI0aanaeaacqaIWaamcqGGUaGlcqaI1aqncqaIWaamcqaIXaqmcqaI3aWnaeaacqaIWaamcqGGUaGlcqaI3aWncqaI4aaocqaI1aqncqaI3aWnaeaacqaIXaqmaeaacqaIWaamcqGGUaGlcqaI2aGncqaI0aancqaIWaamcqaI5aqoaeaacqaIWaamcqGGUaGlcqaIXaqmcqaI2aGncqaIWaamcqaI3aWnaeaacqaIWaamcqGGUaGlcqaIWaamcqaIXaqmcqaIXaqmcqaI3aWnaeaacqaIWaamcqGGUaGlcqaIYaGmcqaI3aWncqaIXaqmcqaI3aWnaeaacqaIWaamcqGGUaGlcqaI0aancqaI2aGncqaIYaGmcqaI0aanaeaacqaIWaamcqGGUaGlcqaI2aGncqaI0aancqaIWaamcqaI5aqoaeaacqaIXaqmaeaacqaIWaamcqGGUaGlcqaIZaWmcqaI1aqncqaI4aaocqaI3aWnaeaacqaIWaamcqGGUaGlcqaIWaamcqaI1aqncqaI0aancqaI5aqoaeaacqaIWaamcqGGUaGlcqaIWaamcqaI0aancqaI4aaocqaIWaamaeaacqaIWaamcqGGUaGlcqaIWaamcqaI5aqocqaI4aaocqaI0aanaeaacqaIWaamcqGGUaGlcqaIXaqmcqaI2aGncqaIWaamcqaI3aWnaeaacqaIWaamcqGGUaGlcqaIZaWmcqaI1aqncqaI4aaocqaI3aWnaeaacqaIXaqmaeaacqaIWaamcqGGUaGlcqaIZaWmcqaI3aWncqaI3aWncqaI3aWnaeaacqaIWaamcqGGUaGlcqaIWaamcqaIWaamcqaIXaqmcqaI1aqnaeaacqaIWaamcqGGUaGlcqaIWaamcqaIWaamcqaIZaWmcqaI0aanaeaacqaIWaamcqGGUaGlcqaIWaamcqaIXaqmcqaIXaqmcqaI3aWnaeaacqaIWaamcqGGUaGlcqaIZaWmcqaI3aWncqaI3aWncqaI3aWnaeaacqaIWaamcqGGUaGlcqaIZaWmcqaI3aWncqaI3aWncqaI3aWnaeaacqaIXaqmaaaacaGLOaGaayzkaaGaeiOla4caaa@E61A@

Thus the overlap graph is connected sufficientely. For *L *= 40 we obtained relative errors of the normalization constants between 10^-4^(highest temperature) and 0.4 (lowest temperature) and similar values for *L *= 400.

**Figure 7:**
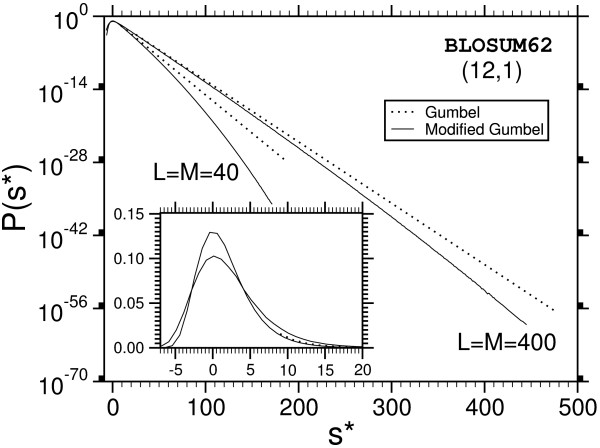
Probability distribution P(s) for gapped sequence alignment using BLOSUM62 matrices and affine gap costs with *α *= 12, *β *= 1 for two sequences lengths *L *= *M *= 40. The results for other lengths are summarized in additional file 1. Strong deviations from the Gumbel distribution become visible in the tail. The dotted lines show the original Gumbel distribution, when fitted to the region of high probability. The inset shows the same data with linear ordinate.

**Figure 8:**
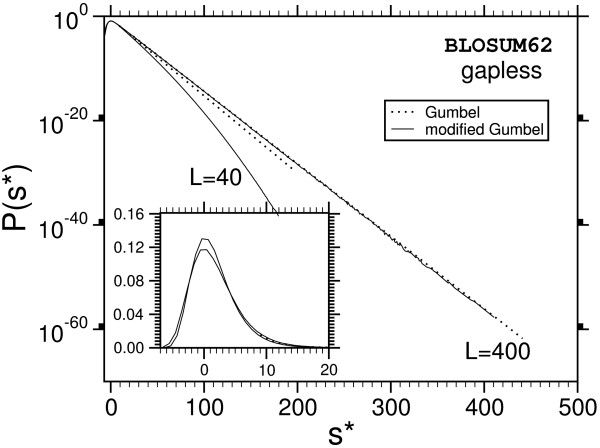
Probability distribution P(s) for ungapped sequence alignment using BLOSUM62-matrices. Deviations form the Gumbel-distribution can only be observed for short sequences (*L *< 250). The inset shows the same data with linear ordinate.

**Figure 9:**
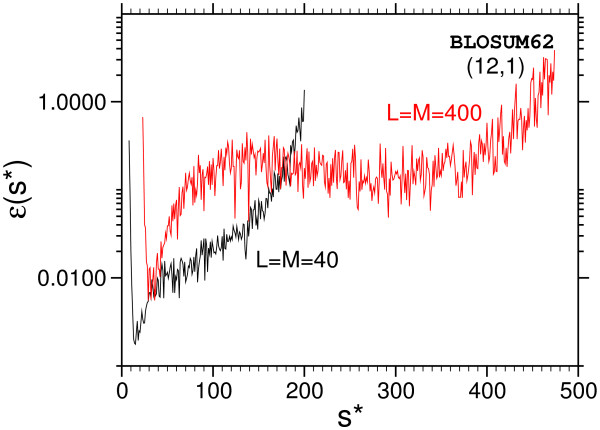
Relative error of the probability estimation using gapped sequence alignment and BLOSUM62 matrices.

The main result is that most of the distributions we obtain deviate strongly from the Gumbel form, which is indicated in Fig. 7 and Fig. 8 by dotted lines. A typical example for the relative error of the results, obtained as explained above, is shown in Fig. 9. Note, that we used normalized scores s* = *s *- *s*_0 _by subtracting the position of the maximum *s*_0 _of the probability distribution. According to Eq. (3), the form of the Gumbel distribution is independent of the sequence length. In the limit *L *= *M *→ ∞. In practice this is not the case due to edge effects [17,18] and database applications use adjusted *λ*'s, but the distribution is still assumed to be of Gumbel form. The results in this work suggest that this is only the case for not too small p-values.

One observes that the discrepancy seems to be stronger for shorter sequences. Also, the case without gaps (Fig. 8) deviates, at least for *L *= *M *= 400, only weakly from the Gumbel distribution. This might be expected due to the previous analytical work [9,10]. Qualitatively the behavior of the PAM250-matrices is the same and therefore the plots are not shown. A quantitative analysis of all results will be given below. Empirically we find that the resulting distribution can be described by a modified Gumbel distribution with a Gaussian correction:

P(s)=PGumbel(s)⋅exp⁡[−λ2(s−s0)2]=λexp⁡[−λ(s−s0)−λ2(s−s0)2−e−λ(s−s0)],
 MathType@MTEF@5@5@+=feaafiart1ev1aaatCvAUfKttLearuWrP9MDH5MBPbIqV92AaeXatLxBI9gBaebbnrfifHhDYfgasaacH8akY=wiFfYdH8Gipec8Eeeu0xXdbba9frFj0=OqFfea0dXdd9vqai=hGuQ8kuc9pgc9s8qqaq=dirpe0xb9q8qiLsFr0=vr0=vr0dc8meaabaqaciaacaGaaeqabaqabeGadaaakeaacqWGqbaucqGGOaakcqWGZbWCcqGGPaqkcqGH9aqpcqWGqbaudaWgaaWcbaGaee4raCKaeeyDauNaeeyBa0MaeeOyaiMaeeyzauMaeeiBaWgabeaakiabcIcaOiabdohaZjabcMcaPiabgwSixlGbcwgaLjabcIha4jabcchaWnaadmaabaGaeyOeI0ccciGae83UdW2aaSbaaSqaaiabikdaYaqabaGccqGGOaakcqWGZbWCcqGHsislcqWGZbWCdaWgaaWcbaGaeGimaadabeaakiabcMcaPmaaCaaaleqabaGaeGOmaidaaaGccaGLBbGaayzxaaGaeyypa0Jae83UdWMagiyzauMaeiiEaGNaeiiCaa3aamWaaeaacqGHsislcqWF7oaBcqGGOaakcqWGZbWCcqGHsislcqWGZbWCdaWgaaWcbaGaeGimaadabeaakiabcMcaPiabgkHiTiab=T7aSnaaBaaaleaacqaIYaGmaeqaaOGaeiikaGIaem4CamNaeyOeI0Iaem4Cam3aaSbaaSqaaiabicdaWaqabaGccqGGPaqkdaahaaWcbeqaaiabikdaYaaakiabgkHiTiabdwgaLnaaCaaaleqabaGaeyOeI0Iae83UdWMaeiikaGIaem4CamNaeyOeI0Iaem4Cam3aaSbaaWqaaiabicdaWaqabaWccqGGPaqkaaaakiaawUfacaGLDbaacqGGSaalaaa@7CA4@

with *s*_0 _= log(*KLM*)/*λ*. Note that we would have to use a different normalization constant here, but since the correction dominates the tail of the distribution, the real normalization constant is numerically indistinguishable from *λ*. We modeled the data by a minimizing a weighted *χ*^2 ^using the program gnuplot [56]. The results including the reduced *χ*^2 ^- values (χ∗2
 MathType@MTEF@5@5@+=feaafiart1ev1aaatCvAUfKttLearuWrP9MDH5MBPbIqV92AaeXatLxBI9gBaebbnrfifHhDYfgasaacH8akY=wiFfYdH8Gipec8Eeeu0xXdbba9frFj0=OqFfea0dXdd9vqai=hGuQ8kuc9pgc9s8qqaq=dirpe0xb9q8qiLsFr0=vr0=vr0dc8meaabaqaciaacaGaaeqabaqabeGadaaakeaaiiGacqWFhpWydaqhaaWcbaGaey4fIOcabaGaeGOmaidaaaaa@3078@ = *χ*^2 ^/degrees of freedom) are documented in Tab. 1 and as an additional CSV-file [see additional file 1].

**Table 1:** Fit parameters of the modified Gumbel distribution Eq. (18) using the BLOSUM62 scoring matrix and affine gap costs with *α *= 10, *β *= 1 . 10^4 ^λ2extra
 MathType@MTEF@5@5@+=feaafiart1ev1aaatCvAUfKttLearuWrP9MDH5MBPbIqV92AaeXatLxBI9gBaebbnrfifHhDYfgasaacH8akY=wiFfYdH8Gipec8Eeeu0xXdbba9frFj0=OqFfea0dXdd9vqai=hGuQ8kuc9pgc9s8qqaq=dirpe0xb9q8qiLsFr0=vr0=vr0dc8meaabaqaciaacaGaaeqabaqabeGadaaakeaaiiGacqWF7oaBdaqhaaWcbaGaeGOmaidabaGaeeyzauMaeeiEaGNaeeiDaqNaeeOCaiNaeeyyaegaaaaa@3671@ describes the estimated value of *λ*_2 _using the scaling relation Eq. (19). Fit parameters for other scoring systems are provided as supplementary material to this artilce [see additional file 1].

*L, M*	*λ*	10^4 ^*λ*_2_	*K*	^*S*^0	χ∗2 MathType@MTEF@5@5@+=feaafiart1ev1aaatCvAUfKttLearuWrP9MDH5MBPbIqV92AaeXatLxBI9gBaebbnrfifHhDYfgasaacH8akY=wiFfYdH8Gipec8Eeeu0xXdbba9frFj0=OqFfea0dXdd9vqai=hGuQ8kuc9pgc9s8qqaq=dirpe0xb9q8qiLsFr0=vr0=vr0dc8meaabaqaciaacaGaaeqabaqabeGadaaakeaaiiGacqWFhpWydaqhaaWcbaGaey4fIOcabaGaeGOmaidaaaaa@3078@	10^4 ^λ2extra MathType@MTEF@5@5@+=feaafiart1ev1aaatCvAUfKttLearuWrP9MDH5MBPbIqV92AaeXatLxBI9gBaebbnrfifHhDYfgasaacH8akY=wiFfYdH8Gipec8Eeeu0xXdbba9frFj0=OqFfea0dXdd9vqai=hGuQ8kuc9pgc9s8qqaq=dirpe0xb9q8qiLsFr0=vr0=vr0dc8meaabaqaciaacaGaaeqabaqabeGadaaakeaaiiGacqWF7oaBdaqhaaWcbaGaeGOmaidabaGaeeyzauMaeeiEaGNaeeiDaqNaeeOCaiNaeeyyaegaaaaa@3671@
40	0.3272 ± 0.108%	8.6347 ± 0.412%	0.1028 ± 0.65%	15.597 ± 0.0676%	79.05	8.1560 ± 12.485%
60	0.3034 ± 0.086%	6.2007 ± 0.285%	0.0751 ± 0.60%	18.455 ± 0.0645%	49.40	6.1711 ± 12.907%
80	0.2892 ± 0.070%	4.8781 ± 0.222%	0.0612 ± 0.53%	20.644 ± 0.0540%	21.67	5.0458 ± 13.280%
100	0.2747 ± 0.072%	4.3187 ± 0.330%	0.0472 ± 0.58%	22.413 ± 0.0611%	39.42	4.3056 ± 13.627%
150	0.2541 ± 0.083%	3.2974 ± 0.529%	0.0303 ± 0.61%	25.682 ± 0.0422%	39.46	3.2047 ± 14.437%
200	0.2432 ± 0.063%	2.6343 ± 0.344%	0.0241 ± 0.52%	28.257 ± 0.0412%	10.47	2.5806 ± 15.214%
250	0.2359 ± 0.071%	2.1999 ± 0.454%	0.0198 ± 0.60%	30.196 ± 0.0459%	9.40	2.1701 ± 15.984%
300	0.2303 ± 0.061%	1.9101 ± 0.348%	0.0174 ± 0.54%	31.934 ± 0.0408%	2.00	1.8758 ± 16.758%
350	0.2261 ± 0.046%	1.6404 ± 0.239%	0.0153 ± 0.41%	33.334 ± 0.0300%	1.27	1.6525 ± 17.544%
400	0.2224 ± 0.052%	1.4806 ± 0.266%	0.0136 ± 0.49%	34.556 ± 0.0369%	1.36	1.4762 ± 18.347%
600	0.2140 ± 0.062%	1.0206 ± 0.384%	0.0106 ± 0.64%	38.561 ± 0.0472%	2.15	1.0250 ± 21.787%
800	0.2090 ± 0.063%	0.7660 ± 0.419%	0.0088 ± 0.67%	41.320 ± 0.0457%	1.82	0.7691 ± 25.697%

All estimated standard errors in this paper are written behind the values and separated by "±".

Note that only for not too small sequences χ∗2
 MathType@MTEF@5@5@+=feaafiart1ev1aaatCvAUfKttLearuWrP9MDH5MBPbIqV92AaeXatLxBI9gBaebbnrfifHhDYfgasaacH8akY=wiFfYdH8Gipec8Eeeu0xXdbba9frFj0=OqFfea0dXdd9vqai=hGuQ8kuc9pgc9s8qqaq=dirpe0xb9q8qiLsFr0=vr0=vr0dc8meaabaqaciaacaGaaeqabaqabeGadaaakeaaiiGacqWFhpWydaqhaaWcbaGaey4fIOcabaGaeGOmaidaaaaa@3078@ is in the order of one. This means that Eq. (18) describes the data better for longer sequences. However biological relevant sequence lengths (*L *> 200) sit in the range were the fit works fine. Moreover the results for shorter sequences are still several orders of magnitude below the naive Gumbel result, which yield χ∗2
 MathType@MTEF@5@5@+=feaafiart1ev1aaatCvAUfKttLearuWrP9MDH5MBPbIqV92AaeXatLxBI9gBaebbnrfifHhDYfgasaacH8akY=wiFfYdH8Gipec8Eeeu0xXdbba9frFj0=OqFfea0dXdd9vqai=hGuQ8kuc9pgc9s8qqaq=dirpe0xb9q8qiLsFr0=vr0=vr0dc8meaabaqaciaacaGaaeqabaqabeGadaaakeaaiiGacqWFhpWydaqhaaWcbaGaey4fIOcabaGaeGOmaidaaaaa@3078@ a value of about 10^4 ^for the *L *= 40 system.

We also tried smaller gap costs than *α *< 10 (*β *= 1, BLOSUM62) and *α *< 11 (*β *= 3, PAM250 matrices), but in this case the distributions deviate from Gumbel not only in the tail but even in the high-probability region. The reason is presumably that the values of the parameters are close to the critical value of the linear-logarithmic phase transition [24], i.e. the alignment is not really local any more.

**Figure 10:**
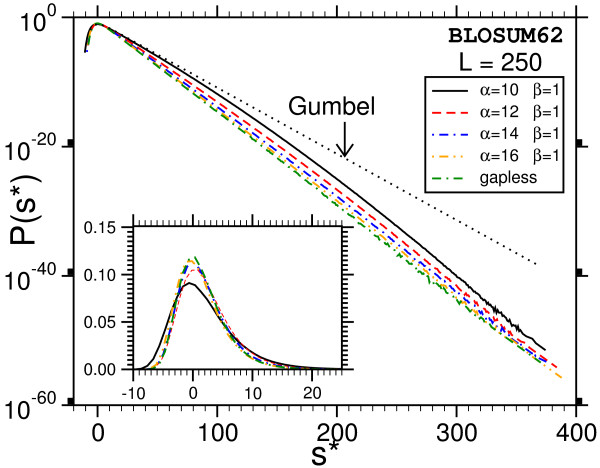
Probability distributions P(s) comparing different gap costs. The dotted line denote the distribution without Gaussian correction (*λ*_2 _= 0). Deviations from the Gumbel distribution become stronger for small gap costs. The inset shows the same data with linear ordinate.

Next, we study the scaling behavior of the correction parameter *λ*_2_. Since the distributions seem to approach the Gumbel distribution with increasing sequence length, as can be seen in Fig. 7 and Fig. 8, we expect that *λ*_2 _decreases for *L *→ ∞. Furthermore, when looking at Fig. 10, where *P *(*s*) is shown for one sequence length *L *= *M *= 250 but for different gap-opening costs *α*, we expect a weak dependence of *λ*_2 _on *α*. In order to provide more quantitative evidence, we fitted all distributions by Eq. (18) and compared the resulting fit parameters.

**Figure 11:**
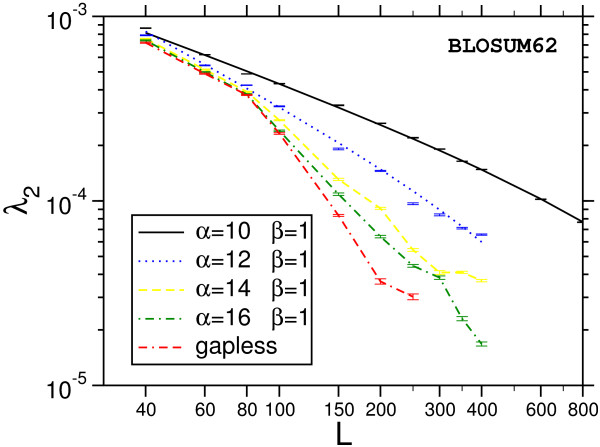
Scaling of the correction parameter *λ*_2 _(BLOSUM62). The decay of *λ*_2 _with system size shows approximately a power law near the logarithm-linear transition (two smallest gap costs). For this cases the fit to Eq. (19) is shown by a line (*α *= 10) and dots (*α *= 12). The lines of the remaining cases are guides to the eye conneting the data points.

**Figure 12:**
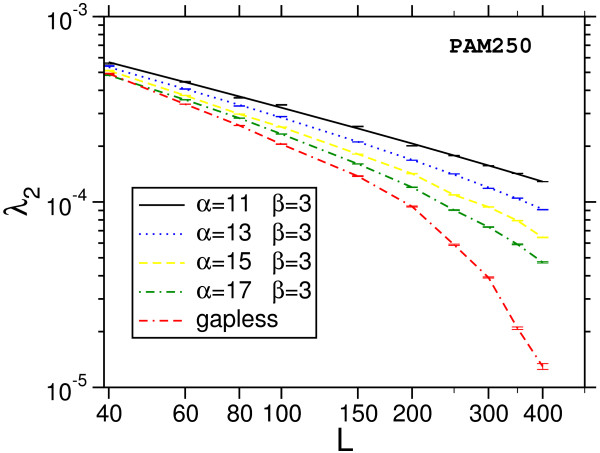
Scaling of the correction parameter *λ*_2 _(PAM250). The decay of *λ*_2 _with system size shows approximately a power law near the logarithm-linear transition (two smallest gap costs). For this cases the fit to Eq. (19) is shown by a line (*α *= 11) and dots (*α *= 13). The lines of the remaining cases are guides to the eye conneting the data points.

In the gapless case no deviations from Gumbel could be detected for sequence lengths *L *> 200. For the other cases, the dependence of the scaling behavior *λ*_2 _on the sequence length is plotted in Fig. 11 and Fig.12. BLOSUM62 and PAM250 behaves qualitatively the same. *λ*_2 _seems to decay with a power law

λ2(L)=a L−b−λ2∗
 MathType@MTEF@5@5@+=feaafiart1ev1aaatCvAUfKttLearuWrP9MDH5MBPbIqV92AaeXatLxBI9gBaebbnrfifHhDYfgasaacH8akY=wiFfYdH8Gipec8Eeeu0xXdbba9frFj0=OqFfea0dXdd9vqai=hGuQ8kuc9pgc9s8qqaq=dirpe0xb9q8qiLsFr0=vr0=vr0dc8meaabaqaciaacaGaaeqabaqabeGadaaakeaaiiGacqWF7oaBdaWgaaWcbaGaeGOmaidabeaakiabcIcaOiabdYeamjabcMcaPiabg2da9iabdggaHjabbccaGiabdYeamnaaCaaaleqabaGaeyOeI0IaemOyaigaaOGaeyOeI0Iae83UdW2aa0baaSqaaiabikdaYaqaaiabgEHiQaaaaaa@3DB6@

for the smallest gap costs and faster than a power law for larger gap costs.

By fitting the limiting cases (two smallest gap costs) to this function an upper bound of the decay could be estimated. The results are summarized in Table 2.

**Table 2:** Fitting parameters of the scaling relation Eq. (19).

Parameter	BLOSUM62 *α *= 10, *β *= 1	BLOSUM62 *α *= 12, *β *= 1
*a*	0.00928 ± 0.0001	0.0309 ± 0.01
*b*	0.643 ± 0.027	0.971 ± 0.08
10^-5 ^λ2∗ MathType@MTEF@5@5@+=feaafiart1ev1aaatCvAUfKttLearuWrP9MDH5MBPbIqV92AaeXatLxBI9gBaebbnrfifHhDYfgasaacH8akY=wiFfYdH8Gipec8Eeeu0xXdbba9frFj0=OqFfea0dXdd9vqai=hGuQ8kuc9pgc9s8qqaq=dirpe0xb9q8qiLsFr0=vr0=vr0dc8meaabaqaciaacaGaaeqabaqabeGadaaakeaaiiGacqWF7oaBdaqhaaWcbaGaeGOmaidabaGaey4fIOcaaaaa@3075@	4.9 ± 1.2	3.2 ± 2.0

Parameter	PAM250 *α *= 11, *β *= 3	PAM250 *α *= 13, *β *= 3

*a*	0.0049 ± 0.0008	0.0053 ± 0.0005
*b*	0.575 ± 0.046	0.591 ± 0.023
10^-5 ^λ2∗ MathType@MTEF@5@5@+=feaafiart1ev1aaatCvAUfKttLearuWrP9MDH5MBPbIqV92AaeXatLxBI9gBaebbnrfifHhDYfgasaacH8akY=wiFfYdH8Gipec8Eeeu0xXdbba9frFj0=OqFfea0dXdd9vqai=hGuQ8kuc9pgc9s8qqaq=dirpe0xb9q8qiLsFr0=vr0=vr0dc8meaabaqaciaacaGaaeqabaqabeGadaaakeaaiiGacqWF7oaBdaqhaaWcbaGaeGOmaidabaGaey4fIOcaaaaa@3075@	3.015 ± 2.0	6.1 ± 1.1

Note that these arguments are purely heuristical attempts to look at the scaling behaviour and its upper bound. It is hard to decide, wether the extrapolation is valid for *L *= *M *→ ∞. However an important range of biological interessting sequence lengths are governed with this scaling analysis.

In order to see the relevance of our result we consider a simple example, the E-value of a pair of sequences of length *L *= 100 using *α *= 12, *β *= 1 gap costs, the BLOSUM62-matrix and the SWISSPROT database [57], which contains currently *N*_swissprot _= 210, 623 sequences. In BLAST [58], the E-value, i.e. the expected number of hits exhibiting at certain "cut-off" score *b*_cut_, is currently estimated via the cumulative Gumbel distribution

E=KLN⋅e−λbcut,
 MathType@MTEF@5@5@+=feaafiart1ev1aaatCvAUfKttLearuWrP9MDH5MBPbIqV92AaeXatLxBI9gBaebbnrfifHhDYfgasaacH8akY=wiFfYdH8Gipec8Eeeu0xXdbba9frFj0=OqFfea0dXdd9vqai=hGuQ8kuc9pgc9s8qqaq=dirpe0xb9q8qiLsFr0=vr0=vr0dc8meaabaqaciaacaGaaeqabaqabeGadaaakeaacqWGfbqrcqGH9aqpcqWGlbWscqWGmbatcqWGobGtcqGHflY1cqWGLbqzdaahaaWcbeqaaiabgkHiTGGaciab=T7aSjabdkgaInaaBaaameaacqqGJbWycqqG1bqDcqqG0baDaeqaaaaakiabcYcaSaaa@3F2D@

where *L *is the query length and *N *the total number of amino acids of the entire database, with parameters *K *= 0.0410 and *λ *= 0.267. Using the suggested E-value of 10 [58], we find a cut-off of *b*_cut _= 64.8 above which a result is considered to be significant, with ℙ [*S *> *b*_cut_] = 4.75 × 10^-5^. Our cumulative distribution achieves this probability at *b*_cut _= 54, i.e. significantly below the BLAST value. Hence, using the true distributions of the scores, a considerable amount of queries, those which have a score between 54 and 64, are significant in contrast to the result of the significance estimation within the Gumbel approximation. Hence, using the data provided in this work, one is able to estimate the significance of protein-data-base queries for the most commonly used parameter sets with much higher precission than when applying the approximation of the Gumbel distribution.

### Sum statistics of the *k*-best alignments

The asymptotic distribution of the ungapped sum statistics is well known by Eq. (5). Again, we are interested in the distributions for *finite *sequence lengths. We use the SIM procedure [27] to compute the sum of the *k*-best alignments (*k *= 2, ..., 5) within the same type of Markov-chain Monte Carlo simulation as in the previous sections. In this case, we consider only the BLOSUM62 matrix together with affine gap costs *α *= 12, *β *= 1, a commonly used scoring system. We observed large fluctuations for short sequences (*L *< 100) and equilibration turned out to be harder for this case. Thus only sequences with *L *≥ 60 (*k *= 2) and *L *≥ 80 (*k *≥ 3) have been used for the analysis. The temperature sets varied between {2.75, 3.0, 3.5, 4.0, 7.0, ∞} for *L *= 100, *k *= 2 and {6.25, 6.5, 7, 9, 11, ∞} for *L *= 400, *k *= 5 (details are shown in Tab. 3).

**Table 3:** Temperature parameters for sum-statistics.

*L*	*k *= 2	*k *= 3	*k *= 4	*k *= 5
40	2.75, 3, 3.5, 4, 7, ∞			
60	2.75, 3, 3.5, 4, 7, ∞			
80	2.75, 3, 3.5, 4, 7, ∞	3.75, 4, 4.5, 5, 8, ∞	5.25, 5.5, 6, 8, ∞	6, 6.25, 6.5, 7, 8, 12, ∞
100	2.75, 3, 3.5, 4, 7, ∞	3.75, 4, 4.5, 5, 8, ∞	5.25, 5.5, 6, 8, ∞	6, 6.25, 6.5, 7, 8, 12, ∞
150	2.75, 3, 3.5, 4, 7, ∞	3.75, 4, 4.5, 5, 8, ∞	5.25, 5.5, 6, 8, ∞	6, 6.25, 6.5, 7, 8, 12, ∞
200	3.25.3.5, 4, 7, ∞	3.75, 4, 4.25, 4.5, 5, 8, ∞	4.75, 5, 5.25, 5.5, 6, 8, ∞	5.75, 6, 6.25, 6.5, 7, 8, 12,∞
300	3.25.3.5, 4, 7, ∞	3.75, 4, 4.25, 4.5, 5, 8, ∞	4.75, 5, 5.25, 5.5, 6, 8, ∞	5.75, 6, 6.25, 6.5, 7, 8, 12,∞
400	3.25.3.5, 3.75, 4, 4.25, 5, 8,∞	3.75, 4, 4.25, 4.5, 5, 8, ∞	5.25, 5, 5.75, 6, 8, 10, ∞	6, 6.25, 6.5, 7, 9, 11,∞

Note that for *k *> 3 the systems could not be equilibrated in the very low temperature regime *T *< 5. Therefore, for theses cases, the tail could only be obtained in an intermediate range of probabilities (~10^-20^), which is nevertheless low enough to obtain significance figures much better compared to using a simple-sampling approach.

**Figure 13:**
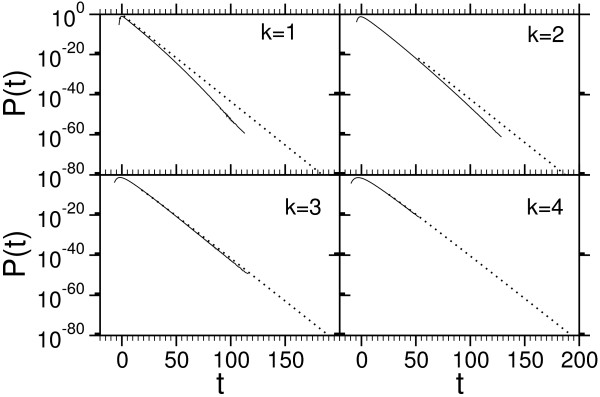
Score probability distributions for sum-statistics of the *k*-best scores (solid lines) for *L *= *M *= 200. The dotted lines denote the distribution without Gaussian correction (*λ*_2 _= 0). Deviations from Eq. (3) or Eq. (6) become only visible in the rare-event tail.

In Fig. 13 we compare different distributions obtained for varying *k *and fixed sequence length *L *= 200. Similar to the case of optimal alignment quadratic deviations could be observed which decrease with growing system length for all values of *k *(not shown).

In order to quantitatively compare the distribution with theoretical predictions from Karlin-Altschul statistics [28], we used the estimated Gumbel parameters *λ *and *s*_0 _from the optimal score distributions. Corresponding to substituting the normalized score in Eq. (6) with *t *= *λ *(*s *- *ks*_0_) we fitted the tail (*p *< 10^-10^) of the Monte Carlo data to the modified distribution of the sum statistics, where the functional form *f*_tail _from Eq. (6) is again modified by a Gaussian factor:

P(s)=Cftail[λ(s−ks0)]⋅exp⁡[λ2(k)(S−kS0)2].
 MathType@MTEF@5@5@+=feaafiart1ev1aaatCvAUfKttLearuWrP9MDH5MBPbIqV92AaeXatLxBI9gBaebbnrfifHhDYfgasaacH8akY=wiFfYdH8Gipec8Eeeu0xXdbba9frFj0=OqFfea0dXdd9vqai=hGuQ8kuc9pgc9s8qqaq=dirpe0xb9q8qiLsFr0=vr0=vr0dc8meaabaqaciaacaGaaeqabaqabeGadaaakeaacqWGqbaucqGGOaakcqWGZbWCcqGGPaqkcqGH9aqpcqWGdbWqcqWGMbGzdaWgaaWcbaGaeeiDaqNaeeyyaeMaeeyAaKMaeeiBaWgabeaakiabcUfaBHGaciab=T7aSjabcIcaOiabdohaZjabgkHiTiabdUgaRjabdohaZnaaBaaaleaacqaIWaamaeqaaOGaeiykaKIaeiyxa0LaeyyXICTagiyzauMaeiiEaGNaeiiCaa3aamWaaeaacqWF7oaBdaqhaaWcbaGaeGOmaidabaGaeiikaGIaem4AaSMaeiykaKcaaOGaeiikaGIaem4uamLaeyOeI0Iaem4AaSMaem4uam1aaSbaaSqaaiabicdaWaqabaGccqGGPaqkdaahaaWcbeqaaiabikdaYaaaaOGaay5waiaaw2faaiabc6caUaaa@5E27@

**Figure 14:**
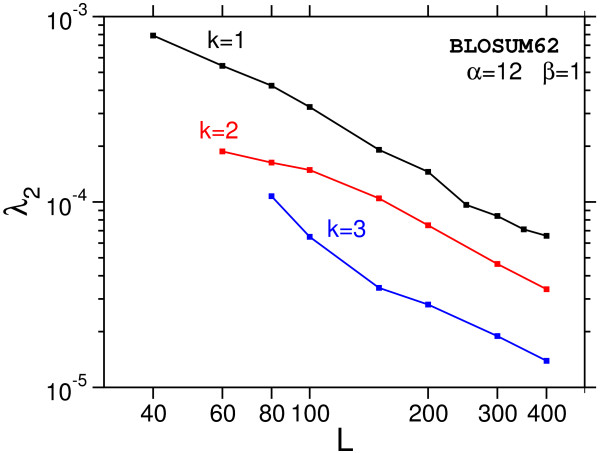
Scaling of the correction parameter for BLOSUM62 sum-statistics (*k *= 1, 2, 3). *λ*_2 _is estimated by a fit for Eq. (21) using optimal the Gumbel-parameters *λ *and *S*_0 _from optimal score statistics (*k *= 1).

This was possible for *k *= 2 and *k *= 3. The results are summarized in Tab. 4 and the scaling behaviour of λ2(k)
 MathType@MTEF@5@5@+=feaafiart1ev1aaatCvAUfKttLearuWrP9MDH5MBPbIqV92AaeXatLxBI9gBaebbnrfifHhDYfgasaacH8akY=wiFfYdH8Gipec8Eeeu0xXdbba9frFj0=OqFfea0dXdd9vqai=hGuQ8kuc9pgc9s8qqaq=dirpe0xb9q8qiLsFr0=vr0=vr0dc8meaabaqaciaacaGaaeqabaqabeGadaaakeaaiiGacqWF7oaBdaqhaaWcbaGaeGOmaidabaGaeiikaGIaem4AaSMaeiykaKcaaaaa@3297@ is shown in Fig. 14. As in the case of the optimal score (*k *= 1), deviations from the theoretical form are significant only in the regime of small probabilities, which is not accessible with naive sampling methods. The data for *k *= 1 to *k *= 3 (Fig. 14) give evidence that the edge effect is reduced by increasing *k*. Note that in Ref. [16], best agreement with theory was achieved with *k *= 6.

**Table 4:** Correction parameter *λ*_2 _for the sum statistics *k *= 2 and *k *= 3. *λ*_2 _is estimated by a fit for Eq. (21) using optimal the Gumbel-parameters *λ *and *S*_0 _from optimal score statistics (*k *= 1). BLOSUM62 with affine gap costs (*α *= 12, *β *= 1) was used as scoring system.

*L*	10^4 ^λ2(k=2) MathType@MTEF@5@5@+=feaafiart1ev1aaatCvAUfKttLearuWrP9MDH5MBPbIqV92AaeXatLxBI9gBaebbnrfifHhDYfgasaacH8akY=wiFfYdH8Gipec8Eeeu0xXdbba9frFj0=OqFfea0dXdd9vqai=hGuQ8kuc9pgc9s8qqaq=dirpe0xb9q8qiLsFr0=vr0=vr0dc8meaabaqaciaacaGaaeqabaqabeGadaaakeaaiiGacqWF7oaBdaqhaaWcbaGaeGOmaidabaGaeiikaGIaem4AaSMaeyypa0JaeGOmaiJaeiykaKcaaaaa@348F@	10^4 ^λ2(k=3) MathType@MTEF@5@5@+=feaafiart1ev1aaatCvAUfKttLearuWrP9MDH5MBPbIqV92AaeXatLxBI9gBaebbnrfifHhDYfgasaacH8akY=wiFfYdH8Gipec8Eeeu0xXdbba9frFj0=OqFfea0dXdd9vqai=hGuQ8kuc9pgc9s8qqaq=dirpe0xb9q8qiLsFr0=vr0=vr0dc8meaabaqaciaacaGaaeqabaqabeGadaaakeaaiiGacqWF7oaBdaqhaaWcbaGaeGOmaidabaGaeiikaGIaem4AaSMaeyypa0JaeG4mamJaeiykaKcaaaaa@3491@
60	2.692 ± 0.30%	
80	1.631 ± 0.63%	1.074 ± 2.59%
100	1.488 ± 0.23%	0.649 ± 2.06%
150	1.056 ± 0.06%	0.344 ± 1.90%
200	0.749 ± 0.13%	0.280 ± 1.14%
300	0.463 ± 0.15%	0.189 ± 0.70%
400	0.338 ± 0.29%	0.139 ± 0.92%

## Discussion and summary

We have studied the distribution of optimum alignment scores over a wide range using a rare-event sampling method. First, by comparing the results for a small 4-letter test system, we illustrated how the method works and provided some evidence for its convergence. In the main part, we considered protein alignment for two types of substitution matrices, i.e. BLOSUM and PAM matrices. We also studied many different sets of biologically relevant parameters by varying gap costs and sequence lengths.

For large enough gap costs it was previously assumed that the distribution follows the Gumbel extreme-value distribution, even when aligning finite sequences and allowing for gaps. Hence, the Gumbel distribution is used for calculating p-values in protein data bases so far. We observe clear deviations from the Gumbel distribution in the biologically relevant rare-event-tail, which is out of reach of simple sampling methods used so far.

An analysis of the scaling behavior of the correction parameter *λ*_2 _gives evidence that the Gumbel distribution correctly describes the data only in the limit of infinite sequence lengths, even for gapped sequence alignments. For finite protein lengths of biological relevance, we observed that the distributions can be fitted well by a Gumbel distribution with a Gaussian correction. Therefore, for data bases like BLAST [8,18,58], we recommend to use distribution functions determined by the empirical fitting parameters provided in this work because the critical value *S*_cut_, above which a result is considered to be significant, changes considerably, as we have seen.

We have also studied the sum-statistics of the *k*-best alignments. Again a Gaussian correction to the assumed form of the distribution was found empirically. Extrapolation to infinitely long sequences gives good evidence that the ungapped statistical theory describes the gapped case for *L *= *M *→ ∞ as well.

## Supplementary Material

Additional file 1 **Fit parameter of the modified Gumbel distribution**. CSV file (tabulator separated) of fit parameters of the modified Gumbel distribution Eq. (18) using different scoring matrices (BLOSUM62 and (PAM250) and gap costs. 10^4 ^λ2extra
 MathType@MTEF@5@5@+=feaafiart1ev1aaatCvAUfKttLearuWrP9MDH5MBPbIqV92AaeXatLxBI9gBaebbnrfifHhDYfgasaacH8akY=wiFfYdH8Gipec8Eeeu0xXdbba9frFj0=OqFfea0dXdd9vqai=hGuQ8kuc9pgc9s8qqaq=dirpe0xb9q8qiLsFr0=vr0=vr0dc8meaabaqaciaacaGaaeqabaqabeGadaaakeaaiiGacqWF7oaBdaqhaaWcbaGaeGOmaidabaGaeeyzauMaeeiEaGNaeeiDaqNaeeOCaiNaeeyyaegaaaaa@3671@ describes the estimated value of *λ*_2 _using the scaling relation Eq. (19) (for small gap costs only). Click here for file

## References

[B1] Brown S (2000). Bioinformatics.

[B2] Rashidi S, Buehler L (2000). Bioinformatics Basics.

[B3] The Protein Data Bank. http://www.pdb.org..

[B4] Fraser C, Gocayne J (1995). The Minimal Gene Complement of Mycoplasma Genitalium. Science.

[B5] Needleman SB, Wunsch CD (1970). A General Method Applicabel to Search for Similarities in the Amino Acid Sequence of two Proteins. J Mol Biol.

[B6] Smith TF, Waterman MS (1981). Identification of Common Molecular Subsequences. J Mol Biol.

[B7] Gotoh O (1982). An Improved Algorithm for Matching Biological Sequences. J Mol Biol.

[B8] Altschul S, Gish W, Miller W, Myers E, Lipman D (1990). Basic Local Alignment Search Tool. J Mol Biol.

[B9] Karlin S, Altschul S (1990). Methods for assessing the statistical significance of molecular sequence features by using general scoring schemes. Proc Natl Acad Sci USA.

[B10] Dembo A, Karlin S, Zeitouni O (1994). Limit Distribution of Maximal Non-Aligned Two-Sequence Segmental Score. Ann Prob.

[B11] Yu Y, Hwa T (2001). Statistical Significance of Probabilistic Sequence Alignment and Related Local Hidden Markov Models. J Comp Biol.

[B12] Yu Y, Bundschuh R, Hwa T, Lässig M, Valeriani A (2002). Statistical Significance and Extreme Ensemble of Gapped Local Hybrid Alignment. Biological Evolution and Statistical Physics.

[B13] Kschischo M, Lässig M, Yu Y (2004). Toward an accurate statistics of gapped alignments. Bull Math Biol.

[B14] Siegmund D, Yakir B (2000). Approximate p-Values for Local Sequence Alignments. Annals of Statistics.

[B15] Metzler D, Grossmann S, Wakolbinger A (2002). A poisson model for gapped local alignments. Stat Prob Letters.

[B16] Altschul S, Gish W (1996). Local Alignment Statistics. Meth Enzym.

[B17] Olsen R, Bundschuh R, Hwa T, Lengauer T, Schneider R, Bork P, Brutlag D, Glasgow J, Mewes HW, Zimmer R, Menlo Park (1999). Rapid Assessment of Extremal Statistics for Local Alignment with Gaps. Proceedings of the seventh International Conference on Intelligent Systems for Molecular Biology.

[B18] Altschul S, Bundschuh R, Olsen R, Hwa T (2001). The estimation of statistical parameters for local alignment score distributions. Nucl Acid Res.

[B19] Hartmann A (2002). Sampling rare events: Statistics of local sequence alignments. Phys Rev E.

[B20] Heinkoff S, Heinkoff J (1992). Amino acid substitution matrices from protein blocks. Proc Natl Acad Sci USA.

[B21] Dayhoff M, Schwartz R, Orcutt B, Dayhoff M (1978). A model of Evolutionary Change in Proteins. Atlas of Protein Sequence and Structure.

[B22] Schwartz R, Dayhoff M, Dayhoff M (1978). Matrices for Detecting Distant Relationships. Atlas of Protein Sequence and Structure.

[B23] Gumbel E (1958). Statistics of Extremes.

[B24] Arratia R, Waterman M (1994). A Phase Transition for the Score in Matching Random Sequences Allowing Deletions. Ann Appl Prob.

[B25] Hwa T, Lässig M, Istrail S, Pevzner P, Waterman M (1998). Optimal Detection of Sequence Similarity by Local Alignment. Proceedings of the Second Annual International Conference on Computational Molecular Biology (RECOMB98).

[B26] Sellers P (1984). Pattern recognition in genetic sequences by mismatch density. Bull Math Biol.

[B27] Altschul S, Erickson B (1986). Locally optimal subalignments using nonlinear similartity functions. Bull Math Biol.

[B28] Karlin S, Altschul S (1993). Applications and statistics for multiple high-scoring segments in molecular sequences. Proc Natl Acad Sci USA.

[B29] Dieker A, Mandjes M (2005). On Asymptotically efficient simulation of large deviation probabilities. Adv Appl Prob.

[B30] Hastings WK (1970). Monte Carlo Sampling Methods Using Markov Chains and Their Applications. Biometrika.

[B31] Liu J (2002). Monte Carlo Strategies in Scientific Computing.

[B32] Liu J (1996). Metropolized independent sampling with comparisons to rejection sampling and importance sampling. Statist Comput.

[B33] Geyer C (1991). Monte Carlo Maximum Likelihood for Depend Data. Proceedings of the 23rd Symposium on the Interface.

[B34] Hukushima K, Nemoto K (1996). Exchange Monte Carlo Method and Application to Spin Glass Simulations. J Phys Soc Jpn.

[B35] Earl D, Deem M (2005). Parallel tempering: Theory, applications, and new perspectives. Phys Chem Chem Phys.

[B36] Zhou R (2004). Exploring the protein folding free energy landscape: Coupling replica exchange method with P3ME/RESPA algorithm. J Molec Graph Mod.

[B37] Zhou R, Berne B (2002). Can a continuum solvent model reproduce the free energy landscape of a *β *-hairpin folding in water?. Proc Natl Acad Sci USA.

[B38] Zhou R, Berne B (2002). Trp-cage: Folding free energy landscape in explicit water. Proc Natl Acad Sci USA.

[B39] Garci'a A, Onuchic J (2003). Folding a protein in a computer: An atomic description of the folding/unfolding of protein. Proc Natl Acad Sci USA.

[B40] Zhou R, Berne B, Germain R (2001). The free energy landscape for *β *hairpin folding in explicit water. Proc Natl Acad Sci USA.

[B41] Auer S, Frenkel D (2001). Prediction of absolute crystal-nucleation rate in hard-sphere colloids. Nature.

[B42] Marinari E, Parisi G, Ruiz-Lorenzo J, Young A (1998). Numerical Simulations of Spin Glass Systems. Spin Glasses and Random Fields, Directions in Condensed Matter Physics.

[B43] Katzgraber H, Palassini M, Young A (2001). Monte Carlo simulations of spin glasses at low temperatures. Phys Rev B.

[B44] Körner M, Katzgraber H, Hartmann A (2006). Probing tails of energy distributions using importance-sampling in the disorder with a guiding function. Stat Mech.

[B45] Wilbur W (1998). Accurate Monte Carlo Estimation of Very Small P-Values In Markov Chains. Comp Stat.

[B46] Geyer C (1994). Estimating Normalization Constants and Reweighting Mixtures in Markov Chain Monte Carlo. Tech Rep 568.

[B47] Meng X, Wong W (1996). Simulating Ratios of Normalization Constants via a Simple Identity: ATheoretical Exploration. Statistica Sinica.

[B48] Raftery A, Lewis S, Bernardo J, Berger J, Dawid A, Smith A (1992). How Many Iterations in the Gibbs Sampler. Bayesian Statistics 4.

[B49] Cowles M, Carlin B (1996). Markov Chain Monte Carlo Convergence Diagnostics: A Comparative Review. JASA.

[B50] StatLib. http://lib.stat.cmu.edu/.

[B51] Coda R package. http://www.r-project.org/.

[B52] Gelman A, Rubin D (1992). Inference from iterative simulation using multiple sequences. Stat Sci.

[B53] Brooks S, Gelman A (1998). General methods for monitoring convergence of iterative simulations. J Comput Graph Stat.

[B54] BEfron (1982). The Jackknife, the Bootstrap and Other Resampling Plans.

[B55] Robinson A, Robinson L (1991). Distribution of glutamine and asparagine residues and their near neighbours in peptides and proteins. Proc Natl Acad Sci USA.

[B56] gnuplot. http://www.gnuplot.info/.

[B57] SWISSPROT. http://www.expasy.org/.

[B58] NCBI BLAST. http://www.ncbi.nlm.nih.gov/BLAST.

